# Mesenchymal stem cell-derived extracellular vesicles accelerate diabetic wound healing by inhibiting NET-induced ferroptosis of endothelial cells

**DOI:** 10.7150/ijbs.97150

**Published:** 2024-06-17

**Authors:** Wei Lu, Xiaoyang Li, Zheyu Wang, Changbo Zhao, Qi Li, Lei Zhang, Shuofei Yang

**Affiliations:** 1 Department of Vascular Surgery, The Quzhou Affliated Hospital of Wenzhou Medical University, Quzhou People's Hospital, No. 100 Minjiang Avenue, Quzhou 324000, China.; 2 Department of Vascular Surgery, Renji Hospital, School of Medicine, Shanghai Jiao Tong University, Pujian Road 160, Shanghai 200127, China.; 3 Department of Vascular Surgery, Yueyang Hospital of Integrated Traditional Chinese and Western Medicine, Shanghai University of Traditional Chinese Medicine, Ganhe Road 110, Shanghai 200437, PR China.

**Keywords:** mesenchymal stem cell-derived extracellular vesicles, neutrophil extracellular traps, diabetic wound healing, ferroptosis, mitochondrial fusion.

## Abstract

Impaired angiogenesis is a major factor contributing to delayed wound healing in diabetes. Dysfunctional mitochondria promote the formation of neutrophil extracellular traps (NETs), obstructing angiogenesis during wound healing. Mesenchymal stem cell-derived extracellular vesicles (MSC-EVs) have shown promise in promoting tissue repair and regeneration in diabetes; however, the precise pathways involved in this process remain unclear. In this study, NET-induced ferroptosis of endothelial cells (ECs) and angiogenesis were assessed in diabetic wound samples from both patients and animal models. *In vitro* and *in vivo* experiments were performed to examine the regulatory mechanisms of NETs in ECs using specific inhibitors and gene-knockout mice. MSC-EVs encapsulating dysfunctional mitochondria were used to trigger mitochondrial fusion and restore mitochondrial function in neutrophils to suppress NET formation. Angiogenesis in wound tissue was evaluated using color laser Doppler imaging and vascular density analysis. Wound healing was evaluated via macroscopic analysis and histological evaluation of the epithelial gap. NET-induced ferroptosis of ECs was validated as a crucial factor contributing to the impairment of angiogenesis in diabetic wounds. Mechanistically, NETs regulated ferroptosis by suppressing the PI3K/AKT pathway. Furthermore, MSC-EVs transferred functional mitochondria to neutrophils in wound tissue, triggered mitochondrial fusion, and restored mitochondrial function, thereby reducing NET formation. These results suggest that inhibiting NET formation and EC ferroptosis or activating the PI3K/AKT pathway can remarkably improve wound healing. In conclusion, this study reveals a novel NET-mediated pathway involved in wound healing in diabetes and suggests an effective therapeutic strategy for accelerating wound healing.

## Introduction

Delayed wound healing is a major complication of diabetes. It is observed in 19-34% of patients with diabetes, increases the risk of amputation, and imposes a heavy economic burden 1. Impaired angiogenesis is considered a key driver of delayed wound healing in diabetes 2. Angiogenesis is closely associated with endothelial cell (EC) homeostasis. Additionally, programmed cell death, such as ferroptosis and apoptosis, or endothelial-mesenchymal transition of ECs is associated with impairment of angiogenesis 3-5. Therefore, therapeutic strategies targeting EC homeostasis hold great promise in promoting angiogenesis and accelerating wound healing in diabetes 6, 7.

During the initial phase of wound healing, neutrophils are recruited to the wound site, where they participate in phagocytosis, degranulation, and the formation of neutrophil extracellular traps (NETs) 8. In 2004, Brinkmann et al. were the first to report that NETs can capture and kill bacteria 9. NETs are intricate structures composed of chromatin filaments and granule proteins. Diabetic wounds exhibit increased NET formation, which potentially contributes to delayed healing 10. However, the precise mechanisms through which NETs influence wound healing remain elusive, and therapeutic strategies targeting NETs to accelerate wound healing in diabetes are unavailable at present.

Stem cells can promote wound healing primarily through their exocrine functional derivatives such as small extracellular vesicles (EVs) 11. Small EVs refer to nano-sized bilayer membrane structures containing microRNAs, lipids, and proteins. They have shown therapeutic potential in various diseases. In contrast to synthetic biomaterials, EVs exhibit substantially improved histocompatibility, permeability and biodegradability, with minimal cytotoxicity 12. In addition, EVs have higher stability, lower immunogenicity, fewer ethical issues, lower risk of embolism, and lower carcinogenicity than their parental cells 13.

In recent years, human umbilical cord mesenchymal stem cell (MSC)-derived extracellular vesicles (MSC-EVs) have attracted increased attention for their ability to promote cutaneous wound healing 14. MSC-EVs inherit potent functions from their parental cells. In particular, they can modulate inflammation and immune responses, stimulate cell proliferation and migration, mitigate oxidative stress, and regulate collagen remodeling. MSC-EVs offer a promising “cell-free” therapeutic approach for chronic wounds, minimizing potential risks associated with direct stem cell transplantation. Although EVs have been shown to play an important role in NET-associated diseases, their use as therapeutic agents has not been evaluated to date 15, 16.

Recent studies have shown that mitochondria, vital organelles supporting cellular functions such as energy production, cellular metabolism, and apoptosis regulation, are involved in the regulation of NETosis 17. Damaged mitochondria generate reactive oxygen species (ROS) and release mitochondrial DNA through the mitochondrial permeability transition pore (mPTP), facilitating the formation of NETs 18-20. Therefore, maintaining mitochondrial integrity in neutrophils can inhibit NET formation 21. MSC-EV-mediated mitochondrial transfer has been shown to modulate the phenotype of macrophages 22. Additionally, MSC-EVs can impede NET formation by restoring mitochondrial function 23. In this study, we evaluated the efficacy of human umbilical cord-derived MSC-EVs in accelerating wound healing in diabetes. We hypothesized that the transfer of functional mitochondria via MSC-EVs might initiate mitochondrial fusion and preserve mitochondrial integrity in neutrophils, consequently inhibiting NET formation and accelerating wound healing.

## Methods

### Clinical data and specimen collection

Participants were divided into four groups as follows: healthy individuals, patients with diabetes, patients with non-diabetic ulcers (NDUs), and patients with diabetic foot ulcers (DFUs). This study was approved by the Institutional Review Board of Renji Hospital, Shanghai Jiao Tong University School of Medicine (Approval No. KY2021-235). All experiments involving human participants strictly adhered to the principles outlined in the Declaration of Helsinki. Before specimen collection, written informed consent was obtained from either the patients or their legal representatives.

### Immunofluorescence staining

For immunofluorescence staining, 5-μm serial sections of paraffin-embedded tissues or primary culture cells were fixed with 4% paraformaldehyde for 15 minutes and permeabilized with 0.1% Triton X-100. Subsequently, the sections or cells were blocked with 1% goat serum at room temperature and incubated with the following primary antibodies (1:200) at 4°C overnight: anti-citH3 (ab5103), anti-CD31 (ab76533), anti-CD31 (ab222783), anti-MPO (ab208670), anti-α-SMA (ab7817), and anti-Ly6g (ab25377) antibodies (Abcam, Boston, USA). The following day, the sections or cells were treated with fluorescently labeled secondary antibodies diluted in a blocking buffer at room temperature for 1 hour. Thereafter, the sections were mounted using 4'6-diamidino-2-phenylindole (Vector ZsBio, Beijing, China), and images were captured using the Leica SP8 confocal microscope (Wetzlar, Germany).

### Isolation and characterization of MSC-EVs

The procedure of MSC-EV isolation was approved by the Institutional Review Board of Renji Hospital, Shanghai Jiaotong University School of Medicine. MSC-EVs were isolated from the supernatants of human umbilical cord-derived MSCs (hUC-MSCs) obtained from the Biotherapy Department of Renji Hospital. The supernatants were sequentially centrifuged at 3000 g (10 minutes, 4°C) and 10,000 g (10 minutes, 4°C). After cellular debris was discarded, MSC-EVs were subjected to two ultracentrifugation steps at 100,000 g (2 h, 4°C) and were subsequently resuspended in PBS. The morphological characteristics of MSC-EVs were examined using transmission electron microscopy (TEM; JEM-1200EX, JEOL, Japan), and their particle size distribution was determined using dynamic light scattering (DLS; Litesizer 500, Anton Paar, Austria). Additionally, western blotting was used to evaluate the expression of EV-associated proteins, including CD9, CD63, CD81, and Alix.

### Assessment of uptake of hUC-MSC-EVs by neutrophils

MSC-EVs were labeled using the PKH26 Cell Membrane Labeling Kit (10-6 M, Sigma-Aldrich, USA) as described in a previous study 24. The cells were cooled to 4°C before their treatment with PBS containing 10% BSA. Neutrophils were isolated from mouse bone marrow and cultured on glass coverslips. PKH26-labeled MSC-EVs were incubated at 37°C for 2 h and fixed with 4% paraformaldehyde (PFA) (pH = 7.4) at room temperature for 30 minutes. Subsequently, the EVs were stained with DAPI for 2 minutes in the dark, and their fluorescence intensity was measured.

### Assessment of ferroptosis markers

The concentration of iron (Fe2+ ions) in cell lysates was measured using an iron assay kit (ab83366; Abcam) according to the manufacturer's instructions. The total concentrations of GSH and glutathione disulfide (GSSG) were determined using a GSSG/GSH quantification kit (G263; Dojindo) according to the manufacturer's instructions. The level of reduced GSH, represented as GSH, was evaluated based on the total glutathione (2GSSG) content, and the GSH-to-GSSG ratio was subsequently calculated. Lipid peroxidation, a crucial indicator assessed by MDA, was analyzed using a lipid peroxidation assay kit (ab118970; Abcam) according to the manufacturer's instructions. The relative concentration of ROS in cell lysates was measured using a ROS assay kit (S0033M; Beyotime) according to the manufacturer's instructions.

### Assessment of mitochondrial function

Mitochondrial DNA (mtDNA) released by neutrophils was quantified as described in a previous study 25. Neutrophils from each experimental group were suspended in digitonin buffer (Millipore, USA) and subjected to agitation at room temperature for 10 minutes. After centrifugation at 16,000 g for 25 minutes at 4°C, the supernatant containing cytosolic mitochondrial DNA (cmtDNA) was collected and the pellet was incubated with a lysis buffer supplemented with proteinase K (Qiagen, USA) and EDTA (5 mM) overnight at 55°C. The following day, the lysate was diluted with double-distilled H_2_O and heated at 95°C for 20 minutes to inactivate proteinase K. The mtDNA present in the pellet, referred to as total mtDNA, was detected using mtDNA-specific primers and SYBR green via quantitative polymerase chain reaction.

The levels of NAD^+^ and NADH in total neutrophil extracts from each group were evaluated using the NAD^+^/NADH Quantification Colorimetric Kit (BioVision, USA) according to the manufacturer's guidelines. The concentration of lactate in neutrophils was measured using the Lactate Colorimetric/Fluorometric Assay Kit (BioVision) according to the manufacturer's protocol. Briefly, neutrophils were washed with lactate assay buffer and incubated with a reaction mixture containing lactate assay buffer, lactate enzyme mix, and lactate probe for 30 minutes at room temperature in the dark. Subsequently, the optical density (OD) of the mixture was measured at a wavelength of 570 nm using a microplate reader (Tecan Spark 10 M).

The MitoProbe (JC1; Life Technologies, NY, USA) was used to assess the mitochondrial electrochemical gradient (ΔΨm) in neutrophils. Neutrophils from each group were collected and incubated with prewarmed JC-1 for 30 minutes at 37°C in the dark. After three washes with prewarmed PBS, the fluorescence intensity was measured using a 13-color FACS Calibur flow cytometer and analyzed using the FlowJo software. The mitochondrial permeability transition pore (mPTP) in neutrophils was examined using the mPTP Fluorescence Assay Kit (Beyotime, China). Briefly, neutrophils were washed twice with prewarmed PBS and stained with calcein AM at 37°C for 45 minutes in the dark. Subsequently, the medium was replaced with a prewarmed culture medium supplemented with 10% FBS, and the cells were incubated at 37°C for 30 minutes in the dark. Finally, nuclei were stained with DAPI, and the cells were observed under a Zeiss 880 confocal microscope.

### Statistical analysis

All data were expressed as the mean ± SD or a percentage. The Kolmogorov-Smirnov test was used to evaluate normality, and non-normally distributed variables were subjected to logarithmic transformation before statistical analysis. The Student's t-test was used to compare continuous variables between groups, whereas one-way ANOVA followed by the Student-Newman-Keuls-q (SNK-q) post hoc test was used for multi-group comparison. The chi-square test was used to compare categorical variables between two groups or among multiple groups. The Gehan-Breslow-Wilcoxon test was used to analyze survival curves. All data were analyzed using the SPSS Statistics software (v22.0; SPSS, Chicago, Illinois, USA), and a P-value of < 0.05 was considered statistically significant.

## Results

### Excessive NET formation hindered diabetic wound healing by affecting angiogenesis

**Figure 1:**
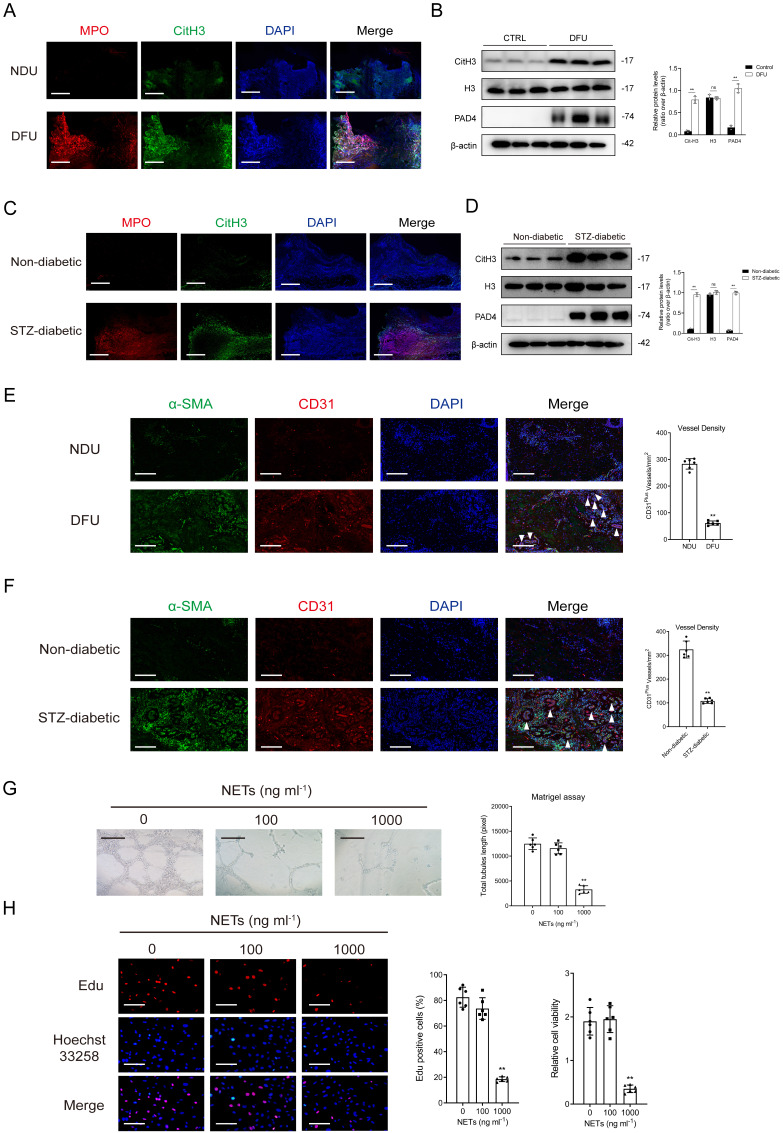
** Excessive NET formation hinders diabetic wound healing by affecting angiogenesis. (A)** Representative images of immunofluorescence staining for MPO (red), Cit-H3 (green), and DAPI (blue) in tissue samples of DFU and controls. Scale bar = 250 µm. **(B)** Western blot for Cit-H3, H3, and PAD4 in tissue samples of DFU vs controls. n = 3, Student's t test. **(C)** Representative images of immunofluorescence staining for MPO (red), Cit-H3 (green), and DAPI (blue) in the tissue samples from the mouse model of diabetic wound and controls. Scale bar = 150 µm. **(D)** Western blot showing Cit-H3, H3, and PAD4 expression in the tissue samples from the mouse model of diabetic wound and controls. N = 3, one-way ANOVA followed by the SNK-q post hoc test. **(E-F)** Left, immunofluorescence staining for CD31 (red) and α-SMA (green) in skin wounds. Right, quantification of vessel density expressed as CD31-positive vessels/mm^2^. scale bar = 100μm. N = 6, Student's t test. **(G)** Representative Matrigel assay images and quantification as total tubule length values. Scale bar = 500μm. N = 6, one-way ANOVA followed by the SNK-q post hoc test. **(H)** Edu and CCK8 assays were performed to evaluate proliferation of ECs treated with or without NETs at low (100 ng/mL) or high (1000 ng/mL) concentration. Scale bar = 50μm. N = 6, one-way ANOVA followed by the SNK-q post hoc test. For all subfigures: *P < 0.05, **P<0.01 compared to other groups.

The proportion of infiltrated neutrophils was higher in DFU tissues than in control tissues in the GSE134431 and GSE199939 datasets (Supplementary Figs. 1A-1B). Gene set enrichment analysis (GSEA) and gene marker analysis revealed that NET formation was significantly higher in DFU tissues (Supplementary Figs. 1C-1F). The results of immunoblotting and immunofluorescence staining indicated that the expression of NET markers was higher in DFU tissues than in control tissues (Figs. 1A-1B). Consistently, mice with diabetic wounds exhibited higher expression of NET markers than control mice (Figs. 1C-1D). Vessel density was significantly lower in the DFU group than in the control group, suggesting impaired angiogenesis in diabetic wounds (Figs. 1E-1F). *In vitro* experiments showed that NET stimulation attenuated the proliferative and tubulogenic abilities of HUVECs (Figs. 1G-1H).

The expression of PAD4 has been reported to be high in diabetes. Here, NETosis was observed in mice with DFUs. Macroscopic analysis and histological evaluation revealed a substantial improvement in wound closure in *Padi4*^-/-^ mice with diabetes (inhibition of NETosis) when compared with the delayed wound closure observed in wild-type mice with diabetes (Supplementary Figs. 2A-2B). The time required for complete wound healing was notably longer in wild-type mice with diabetic wounds than in *Padi4*^-/-^ mice with diabetic or non-diabetic wounds. Doppler analysis showed that wound tissue perfusion and angiogenesis were significantly impaired in mice with diabetes when compared with mice without diabetes. However, downregulation of PAD4 notably enhanced wound tissue perfusion and vessel density (Supplementary Figs. 2C-2D).

### NET-induced ferroptosis of ECs impaired angiogenesis by inhibiting the PI3K/AKT pathway

On analyzing single-cell RNA sequencing data from the GSE165816 dataset, we found that the number of ECs was significantly lower in patients with DFUs than in healthy individuals (Supplementary Figs. 3A-3B). GSEA and gene marker analysis showed significant EC ferroptosis in the DFU group (Supplementary Figs. 3C-3E). The number of cluster 3 ECs was significantly higher in patients with diabetes with non-healing wounds than in those without non-healing wounds (Supplementary Figs. 4A-4B). Furthermore, active cell-cell adhesion was observed in ECs of non-healing diabetic wounds (Supplementary Fig. 4C). The levels of *CXCL*12, a chemokine involved in neutrophil activation, and ferroptosis-related markers were significantly higher in cluster 3 ECs of non-healing diabetic wounds than in those of healing diabetic wounds (Supplementary Figs. 4D-4F). The activity of the PI3K/AKT pathway was significantly lower in ECs of non-healing diabetic wounds than in those of healing diabetic wounds (Supplementary Figs. 5A). In addition, the PI3K/AKT pathway was found to be closely related to NET formation or EC ferroptosis in non-healing diabetic wounds (Supplementary Figs. 5B-5F).

**Figure 2:**
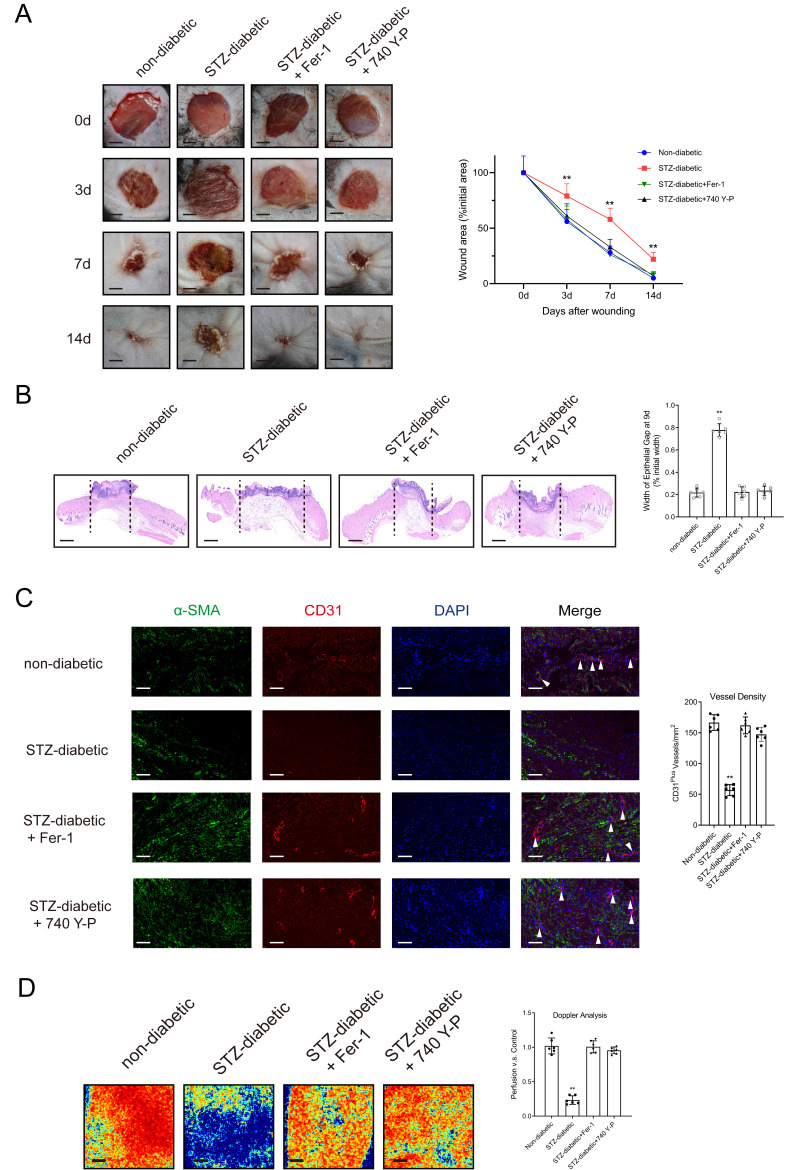
** Activating the PI3K/AKT pathway and inhibiting the ferroptosis of ECs can promote diabetic wound healing. (A)** Left, representative images of wounds at 0, 3, 7, and 14 d post-wound injury in non-diabetic, STZ-diabetic, STZ-diabetic+Fer-1, and STZ-diabetic+740 Y-P groups. Right, level of wound closure is expressed as a percentage of wound area from the initial wound area. Scale bar = 500μm. N = 3, Chi square test. **(B)** Epithelial gap of wound healing on histology was evaluated in non-diabetic, STZ-diabetic, STZ-diabetic + Fer-1, and STZ-diabetic + 740 Y-P groups. The distance between the leading edges was calculated. Scale bar = 100μm. N = 6, one-way ANOVA followed by the SNK-q post hoc test. **(C)** Left, immunofluorescence staining for CD31 and a-SMA of skin wounds (400 ×) in non-diabetic, STZ-diabetic, STZ-diabetic+Fer-1, and STZ-diabetic+740 Y-P groups. Right, quantification of vessel density expressed as CD31-positive vessels/mm^2^. N = 6, one-way ANOVA followed by the SNK-q post hoc test. **(D)** Representative color laser Doppler images taken at 5 days post-wounding. The wound perfusion was calculated as the ratio between treated and control blood flow in non-diabetic, STZ-diabetic, STZ-diabetic+Fer-1, and STZ-diabetic+740 Y-P groups. N = 6, one-way ANOVA followed by the SNK-q post hoc test. For all subfigures: *P < 0.05, **P<0.01 compared to other groups.

HUVECs were treated with varying concentrations of NETs (0-1000 ng/mL) derived from neutrophils activated with phorbol-12-myristate-13-acetate (PMA) for 24 hours. Subsequently, cellular levels of the ferroptosis-related markers GPX4, ACSL4, TFR1, and SLC7A11 were assessed. The results showed that stimulation with NETs decreased the expression of GPX4 and SLC7A11 and increased the expression of ACSL4 and TFR1 in HUVECs in a dose-dependent manner (Supplementary Fig. 6A). Treatment with Fer-1 (an inhibitor of ferroptosis) or DNase I, which degraded NET-DNA, suppressed NET-induced ferroptosis (Supplementary Figs. 6B-6C) and enhanced the proliferative and tubulogenic abilities of NET-treated HUVECs (Supplementary Figs. 6D-6E). Furthermore, stimulation with NETs resulted in a dose-dependent reduction in the expression of PI3K and p-AKT (Supplementary Fig. 6F). On the contrary, treatment with 740 Y-P (a PI3K activator) or DNase I suppressed NET-induced ferroptosis and upregulated the PI3K/AKT pathway (Supplementary Figs. 6F-6H). Stimulation with NETs triggered ferroptosis-related processes, including upregulation of Fe2+ ions, depletion of GSH, lipid peroxidation, and accumulation of MDA. However, treatment with DNase I, Fer-1, or 740 Y-P counteracted the effects of NETs (Supplementary Figs. 7A-7E) 26. Macroscopic analysis and histological evaluation showed that treatment with Fer-1 and 740 Y-P accelerated wound healing (Figs. 2A-2B) and significantly improved wound tissue perfusion and vessel density (Figs. 2C-2D) in mice with diabetes.

### MSC-EVs accelerated diabetic wound healing by inhibiting NET formation

hUC-MSCs were isolated and cultured *in vitro*. Immunostaining revealed the presence of CD105, CD73, CD90, and CD166 but the absence of CD45 and CD34 on the cell surface (Supplementary Fig. 8A). Nanoparticle tracking analysis (NTA) and TEM showed that MSC-EVs had an average size of 125.7 nm and a volume of 134.9 nm³, featuring a distinct ultrastructure resembling a "saucer" (Supplementary Fig. 8B). In addition, western blotting validated the enrichment of exosomal markers such as CD9, CD63, and apoptosis-linked gene 2-interacting protein X (Alix) in MSC-EVs (Supplementary Figs. 8C-8D).

**Figure 3:**
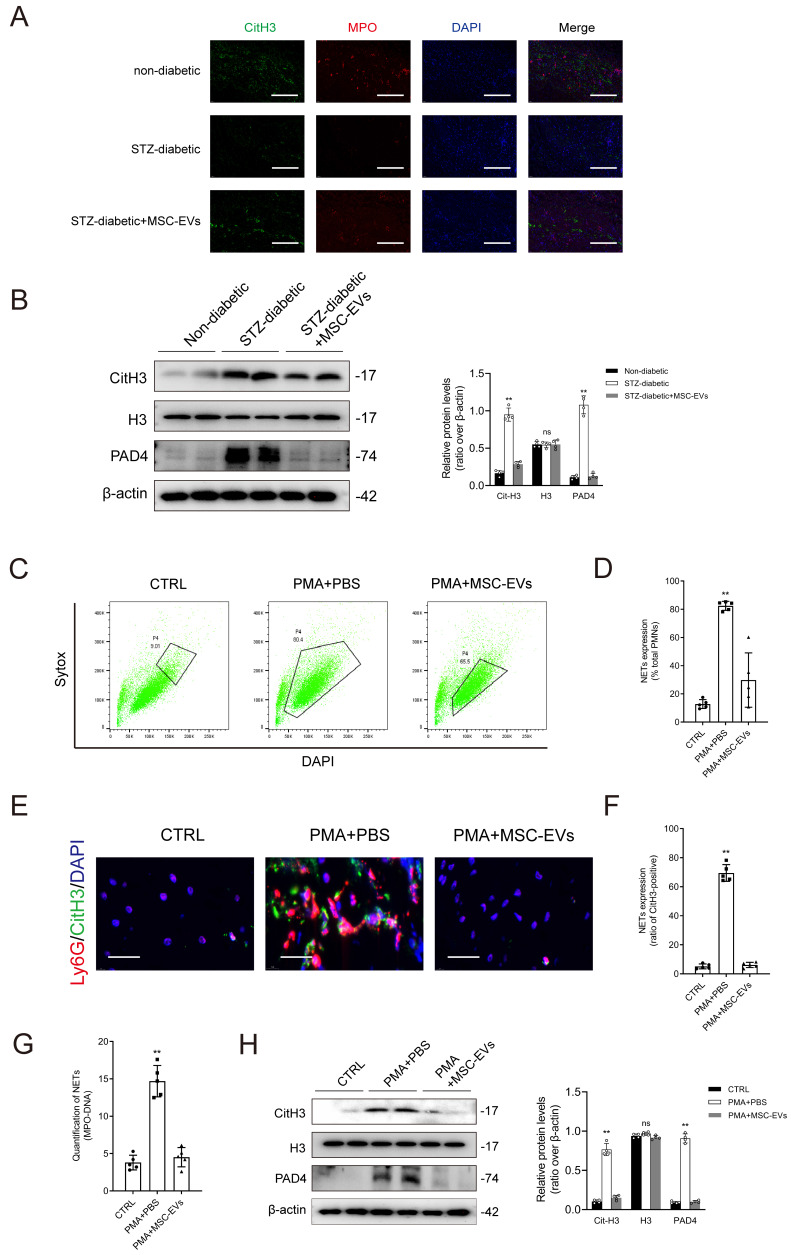
** MSC-EVs inhibit NET formation *in vivo* and *in vitro.* (A)** Representative images showing immunofluorescence staining for MPO (red), CitH3 (green), and DAPI (blue) in the tissue samples from non-diabetic, STZ-diabetic and STZ-diabetic + MSC-EVs groups. Scale bar = 150 µm. **(B)** Western blot showing Cit-H3, H3, and PAD4 expression in the tissue samples from non-diabetic, STZ-diabetic and STZ-diabetic + MSC-EVs groups. N = 4, one-way ANOVA followed by the SNK-q post hoc test. **(C-D)** Flow cytometry analysis of Sytox Green^+^ DAPI^+^ neutrophils after 2 h of PMA treatment. N = 5, one-way ANOVA followed by the SNK-q post hoc test. **(E-F)** Representative immunofluorescence images of MPO (red), CitH3 (green), and DAPI (blue) staining of neutrophils incubated with PBS, PMA, PMA + MES-EVs for 24 h. Scale bar = 50 μm. N = 5, one-way ANOVA followed by the SNK-q post hoc test. **(G)** Quantification of MPO-DNA complexes in the culture supernatant. N = 5, one-way ANOVA followed by the SNK-q post hoc test. **(H)** Western blot showing Cit-H3, H3, and PAD4 in neutrophils treated with PBS, PMA, PMA + MES-EVs. N = 4, one-way ANOVA followed by the SNK-q post hoc test. For all subfigures: *P < 0.05, **P<0.01 compared to other groups.

Immunofluorescence staining and western blotting demonstrated that treatment with MSC-EVs effectively suppressed NET formation in mice with diabetic wounds (Figs. 3A-3B). Sytox Green (SG), a dye used to label plasma membrane-impermeable DNA to quantify NET-DNA, was used for flow cytometry. The results of flow analysis showed that treatment with PMA for 2 hours increased the proportion of SG+DAPI+ neutrophils, whereas pretreatment with MSC-EVs had the opposite effect (Figs. 3C-3D). Immunofluorescence staining showed that MSC-EVs significantly inhibited PMA-induced NET formation (Figs. 3E-3F). Treatment with PMA increased the levels of MPO-DNA complexes in the culture supernatant, whereas pretreatment with MSC-EVs effectively reversed this change (Fig. 3G). Western blotting indicated that MSC-EVs remarkably attenuated the PMA-induced upregulation of citH3 and PAD4 in neutrophils (Fig. 3H). Altogether, these findings validated that MSC-EVs inhibited NET formation both in vitro and in vivo. Furthermore, macroscopic analysis and histological evaluation showed that systemic administration of MSC-EVs accelerated wound healing in mice with diabetic wounds (Supplementary Figs. 9A-9B). Consistently, treatment with Fer-1 and 740 Y-P significantly improved wound tissue perfusion and vessel density in mice with diabetic wounds (Supplementary Figs. 9C-9D).

### MSC-EVs regulated NET formation by transferring functional mitochondria

As shown in Supplementary Figs. 10A-10B, DiR-labeled MSC-EVs were internalized by neutrophils in the wound tissues of mice with diabetes; however, this phenomenon was not observed in peripheral blood samples. PKH26-labeled MSC-EVs were incubated with neutrophils for 2 hours to evaluate their cellular uptake. The results indicated that MSC-EVs were successfully internalized by neutrophils as evidenced by their localization in the cells (Supplementary Fig. 10C).

**Figure 4:**
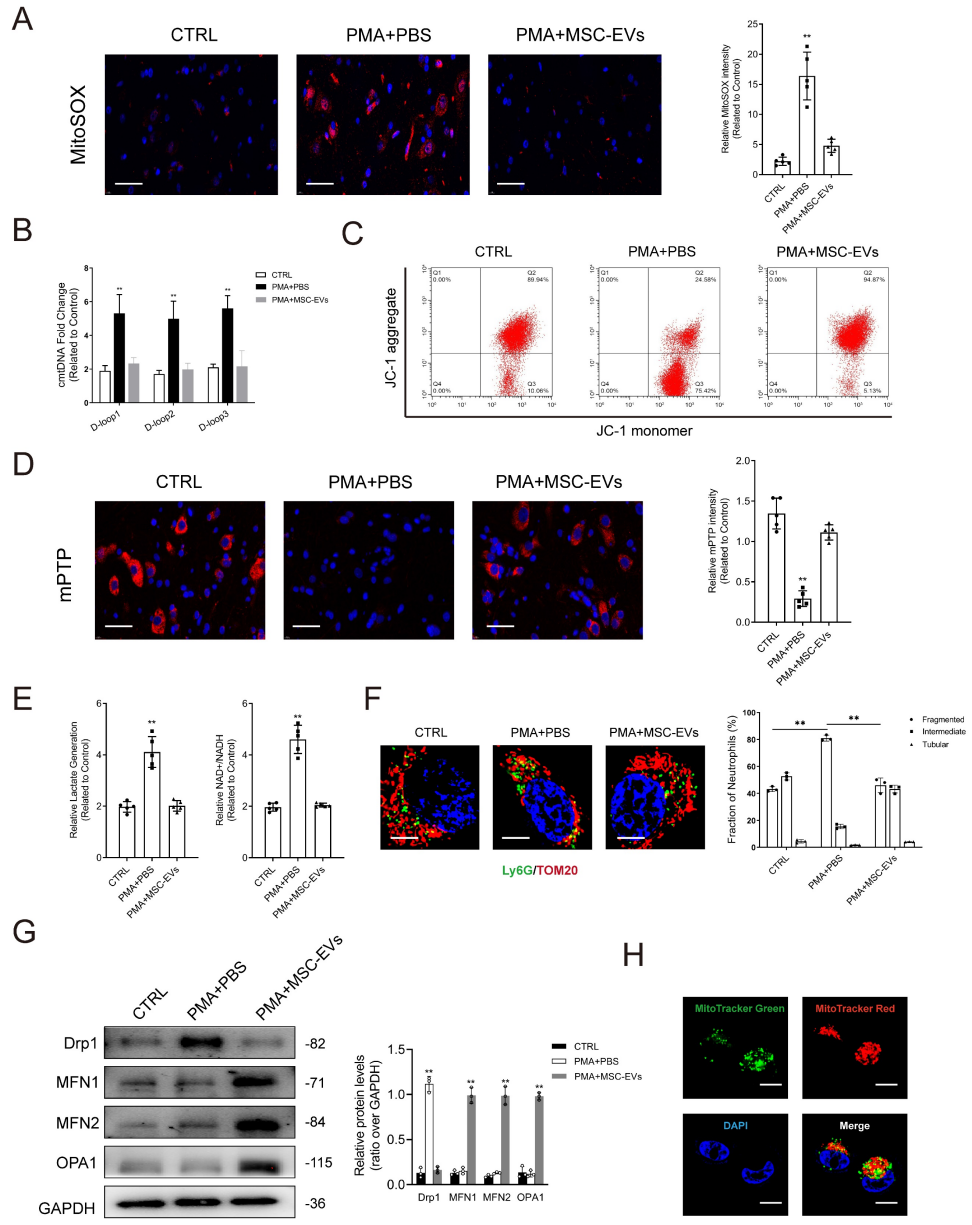
** MSC-EVs transfer functional mitochondria to neutrophils to induce mitochondrial fusion and enhance mitochondrial function in neutrophils. (A-B)** Representative immunofluorescence images and quantification of mitochondrial ROS (mtROS) and mtDNA into the cytoplasm (cmtDNA) generation in neutrophils treated with PBS, PMA, PMA + MES-EVs. Scale bar = 50 μm. N = 5, one-way ANOVA followed by the SNK-q post hoc test. **(C)** Quantification of mitochondrial membrane potential by using JC-1 (mitoProbe) in neutrophils treated with PBS, PMA, PMA + MES-EVs. **(D)** Representative immunofluorescence images and quantification of mitochondrial permeability transition pore (mPTP) assay in neutrophils treated with PBS, PMA, PMA + MES-EVs. Scale bar = 50 μm. N = 5, one-way ANOVA followed by the SNK-q post hoc test**. (E)** Quantification of lactate production and NAD^+^/NADH ratio in the neutrophils. N = 5, one-way ANOVA followed by the SNK-q post hoc test. **(F)** Representative immunofluorescence imaging and quantification of mitochondria morphology change in the neutrophils treated with PBS, PMA, PMA + MES-EVs. Scale bar = 5 μm. N = 3, one-way ANOVA followed by the SNK-q post hoc test. **(G)** Western blot showing MFN1, MFN2, OPA1, Drp1 expression in neutrophils. N = 3, one-way ANOVA followed by the SNK-q post hoc test. (H) Representative immunofluorescence imaging of MSC-EVs transferring functional mitochondria (Red) to neutrophils (Green). The mitochondria in MSC-EVs integrated into the neutrophils mitochondrial network (Yellow). Scale bar = 5 μm. For all subfigures: *P < 0.05, **P<0.01 compared to other groups.

Impairment of mitochondria contributes to the initiation of NET formation by stimulating ROS production in mitochondria. Simultaneously, it leads to the release of mitochondrial DNA (mtDNA) into the cytoplasm through mPTP. Lactate accumulation is another major factor contributing to NET formation, given the limited presence of mitochondria in neutrophils and their reliance on glycolysis as the primary energy source 27. As shown in Figs. 4A-4B, PMA stimulated mitochondrial ROS (mtROS) generation and cytosolic mitochondrial DNA (cmtDNA) release in neutrophils. JC-1 was used to evaluate the mitochondrial membrane potential (Δ*Ψm)*. The results showed that Δ*Ψm* was imbalanced in neutrophils in the PMA-treated group when compared with those in the control group (Fig. 4C). Treatment with PMA significantly induced the opening of mPTP in neutrophils (Fig. 4D), whereas pretreatment with MSC-EVs reduced mtROS levels, restored Δ*Ψm* balance, and limited mPTP opening, consequently repairing mitochondrial damage in neutrophils (Figs. 4A-4D). Furthermore, changes in oxidative phosphorylation were monitored to assess mitochondrial function in neutrophils. Lactate levels and the NAD+/NADH ratio were higher in PMA-treated neutrophils than in control neutrophils; however, pretreatment with MSC-EVs effectively reversed these changes (Fig. 4E). Mitochondria exhibited a more elongated morphology in MSC-EV-treated neutrophils than in control neutrophils. This phenomenon can be attributed to the transport of functional mitochondria from MSC-EVs to neutrophils, which potentially triggered fusion between functional mitochondria released by MSC-EVs and damaged mitochondria in neutrophils, thereby restoring mitochondrial function (Fig. 4F). Furthermore, we quantified the expression of key factors regulating mitochondrial dynamics, including fusion (OPA1, MFN1, and MFN2) and fission (Drp1) proteins. Pretreatment with MSC-EVs significantly increased the expression of all three fusion regulators, which may explain the elongated mitochondrial morphology (Fig. 4G). MitoTracker Deep Red was used to stain mitochondria in MSC-EVs, whereas MitoTracker Green was used to stain mitochondria in neutrophils. As shown in Fig. 4H, functional mitochondria were successfully transferred from MSC-EVs to neutrophils.

### Inhibition of mitochondrial function attenuated the regulatory effects of MSC-EVs on mitochondrial quality and NET formation

Rhodamine 6G was used to inhibit mitochondrial respiration in MSCs as described in a previous study. Subsequently, purified MSC-EVs encapsulating damaged mitochondria (Rho-EVs) were used to validate the modulatory effects of mitochondria in MSC-EVs on NET formation 28.

**Figure 5:**
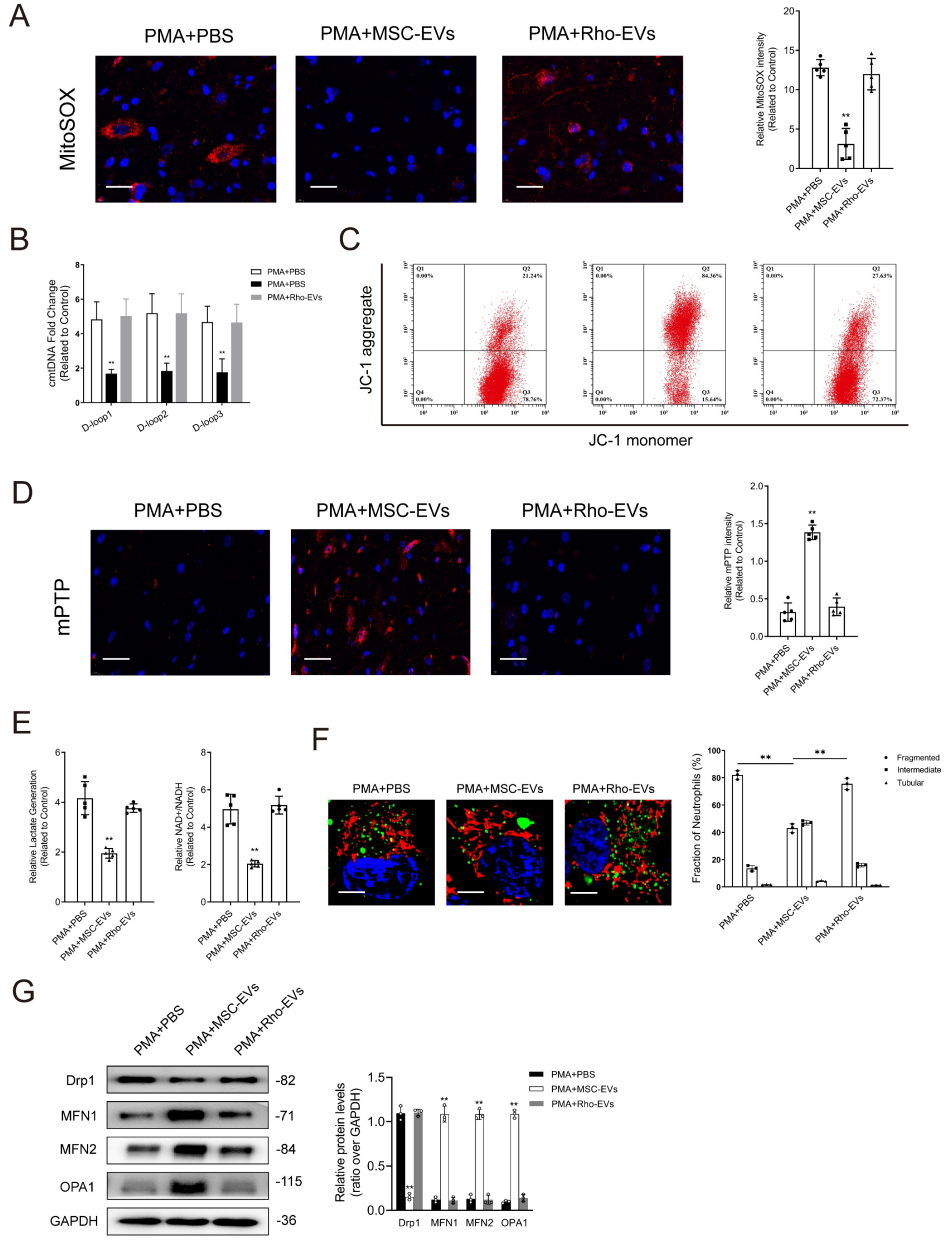
** Impairment of mitochondrial function attenuates the protective effects of MSC-EVs on mitochondrial quality and function in neutrophils. (A-B)** Representative immunofluorescence images and quantification of mitochondrial ROS (mtROS) and mtDNA into the cytoplasm (cmtDNA) generation in neutrophils treated with PMA + PBS, PMA + MES-EVs, and PMA + Rho-EVs. Scale bar = 50 μm. N = 5, one-way ANOVA followed by the SNK-q post hoc test. **(C)** Quantification of mitochondrial membrane potential by using JC-1 (mitoProbe) in neutrophils treated with PMA + PBS, PMA + MES-EVs, and PMA + Rho-EVs. **(D)** Representative immunofluorescence images and quantification of mitochondrial permeability transition pore (mPTP) assay in neutrophils treated with PMA + PBS, PMA + MES-EVs, and PMA + Rho-EVs. Scale bar = 50 μm. N = 5, one-way ANOVA followed by the SNK-q post hoc test. **(E)** Quantification of lactate production and NAD^+^/NADH ratio in the neutrophils treated with PMA + PBS, PMA + MES-EVs, and PMA + Rho-EVs. N = 5, one-way ANOVA followed by the SNK-q post hoc test. **(F)** Representative immunofluorescence imaging and quantification of mitochondria morphology change in the neutrophils treated with PMA + PBS, PMA + MES-EVs, and PMA + Rho-EVs. Scale bar = 5 μm. N = 3, one-way ANOVA followed by the SNK-q post hoc test. **(G)** Western blot showing MFN1, MFN2, OPA1, Drp1 expression in neutrophils treated with PMA + PBS, PMA + MES-EVs, and PMA + Rho-EVs. N = 3, one-way ANOVA followed by the SNK-q post hoc test. For all subfigures: *P < 0.05, **P<0.01 compared to other groups.

The results of NTA and TEM showed that Rho-EVs had similar characteristics to EVs (Supplementary Figs. 11A-11B). Rho-EVs exhibited high levels of EV-related markers but not those of TOM20 (Supplementary Figs. 11C-11D). Treatment with MSC-EVs successfully reduced mtROS generation and cmtDNA accumulation, stabilized ΔΨm, and prevented the opening of mPTP. On the contrary, treatment with Rho-EVs impaired mitochondrial function, partially attenuating the effects of MSC-EVs on neutrophils (Figs. 5A-5D). Treatment with Rho-EVs increased lactate production and the NAD^+^/NADH ratio, indicating that impaired mitochondrial respiration attenuated the ability of MSC-EVs to drive the transition from glycolysis to oxidative phosphorylation in neutrophils (Fig. 5E). In addition, the degree of mitochondrial fusion was notably lower in the Rho-EV group than in the MSC-EV group (Figs. 5F-5G).

Treatment with Rho-EVs partially counteracted the suppressive effects of MSC-EVs on NET formation (Supplementary Figs. 12A-12C). As shown in Supplementary Fig. 12D, treatment with MSC-EVs increased the abundance of MPO-DNA complexes in the culture supernatants of neutrophils, whereas treatment with Rho-EVs reversed this increase.

### Impairment of mitochondrial function counteracted the MSC-EV-induced inhibition of NET formation and acceleration of diabetic wound healing

**Figure 6:**
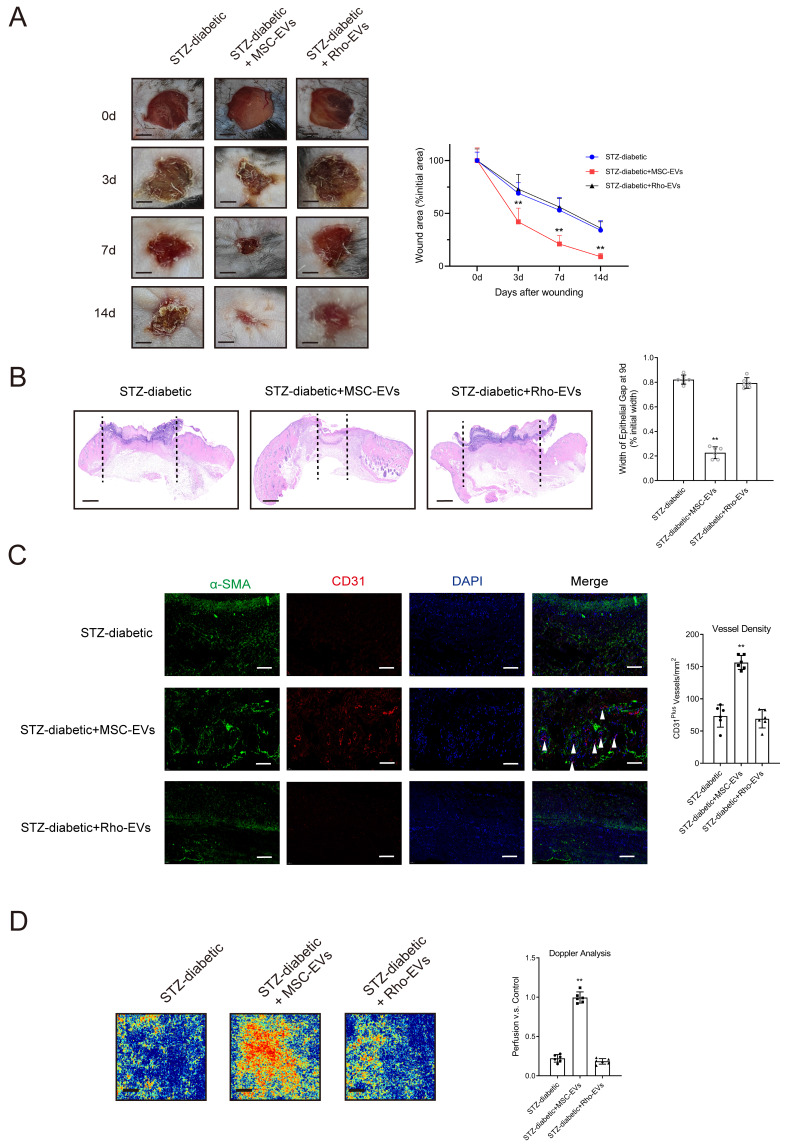
** Impairment of mitochondrial function counteracts the MSC-EV-induced promotion of angiogenesis and acceleration of diabetic wound healing. (A)** Left, representative images of wounds at 0, 3, 7, and 14 d post-wound injury in STZ-diabetic, STZ-diabetic + MSC-EVs, and STZ-diabetic + Rho-EVs groups. Right, level of wound closure is expressed as a percentage of wound area from the initial wound area. Scale bar = 500μm. N = 3, Chi square test. **(B)** Epithelial gap of wound healing on histology was evaluated in STZ-diabetic, STZ-diabetic + MSC-EVs, and STZ-diabetic + Rho-EVs groups. The distance between the leading edges was calculated. Scale bar = 100μm. N = 6, one-way ANOVA followed by the SNK-q post hoc test. **(C)** Left, immunofluorescence staining for CD31 and a-SMA of skin wounds (400 ×) in STZ-diabetic, STZ-diabetic + MSC-EVs, and STZ-diabetic + Rho-EVs groups. Right, quantification of vessel density expressed as CD31-positive vessels/mm^2^. N = 6, one-way ANOVA followed by the SNK-q post hoc test. **(D)** Representative color laser Doppler images taken at 5 days post-wounding. The wound perfusion was calculated as the ratio between treated and control blood flow in STZ-diabetic, STZ-diabetic + MSC-EVs, and STZ-diabetic + Rho-EVs groups. N = 6, one-way ANOVA followed by the SNK-q post hoc test. For all subfigures: *P < 0.05, **P<0.01 compared to other groups.

The degree of NET formation was evaluated in wound tissues. Deterioration of mitochondrial function attenuated the suppressive effects of MSC-EVs on NET formation in mice with diabetic wounds (Supplementary Figs. 13A-13B). Rho-EVs were administered to mice with diabetic wounds to verify whether MSC-EVs improved wound healing via mitochondrial transfer. Histological evaluation showed that treatment with Rho-EVs significantly increased the wound area and epithelial gap of the healing wound, indicating that inhibition of mitochondrial respiration by rhodamine 6G counteracted the MSC-EV-induced acceleration of diabetic wound healing (Figs. 6A-6B). Administration of MSC-EVs improved tissue perfusion and vessel density in mice with diabetic wounds, whereas administration of Rho-EVs reversed the effects of MSC-EVs.

## Discussion

This study revealed a novel NET-related mechanism involved in impairment of angiogenesis during wound healing in diabetes and suggested a promising therapeutic strategy targeting NETs through MSC-EVs. The findings suggested that enhanced NET formation in diabetic wounds induced EC ferroptosis by inhibiting the PI3K/AKT pathway. MSC-EV-mediated transfer of functional mitochondria and induction of mitochondrial fusion inhibited NET formation, accelerating wound healing in diabetes.

Several studies have highlighted the involvement of NETs in the pathogenesis of various diseases, such as cancer, autoimmune conditions, venous thromboembolism, and COVID-19 29-31. Diabetic wounds have an excessive accumulation of NETs. We have previously shown that excessive NET formation serves as a marker of impaired wound healing in patients with DFUs 32. However, the precise mechanisms through which NETs influence diabetic wound healing remain elusive. NET-mediated activation of the NLRP3 inflammasome results in poor angiogenesis and wound closure in diabetes 33. ECs play an essential role in angiogenesis in the wound tissue. Recent studies have shown that NETs can induce the ferroptosis of intestinal endothelial cells, which may result in intestinal microcirculatory dysfunction 34. This study showed that excessive NET formation significantly promoted EC ferroptosis by inhibiting the PI3K/AKT pathway. The underlying mechanism may rely on the effects of NET-derived DNA, as treatment with DNase I counteracted the effects of NETs on ferroptosis. Previous studies have shown that the NET-DNA receptor activates the integrin-linked kinase and PI3K/AKT pathways to promote cutaneous wound healing 31, 35. However, the receptor that mediates NET-induced suppression of the PI3K/AKT pathway in ECs remains unknown. Further investigation is warranted to identify distinct components of NETs and determine the intracellular signal transduction pathways associated with EC ferroptosis.

NETs have been shown to induce ferroptosis in various cell types. In particular, NETs play a role in activating ferroptosis in alveolar epithelial cells by modulating N6-methyladenosine modification 36. Notably, myeloperoxidase, a key component of NETs originating from neutrophil-specific granules, directly induces ferroptosis in glioblastoma cells 37. However, the mechanisms involved in NET-induced ferroptosis of ECs in diabetic wounds remain poorly understood. This study reveals that the PI3K/AKT pathway mediates NET-induced ferroptosis of ECs. Moreover, it implicates the involvement of VEGFR2/PI3K/AKT pathway in diabetes-related impairment of ischemia-mediated angiogenesis 38. Inhibition of ferroptosis by rescuing the PI3K/AKT pathway alleviates inflammatory cell infiltration in diabetic wounds and accelerates wound healing 39.

As PI3K serves as a crucial mediator of ferroptosis, PI3K inhibitors represent promising agents for inducing immunogenic ferroptosis 40. This study revealed that the PI3K agonist 740 Y-P effectively suppressed EC ferroptosis, accelerating wound healing in mice with diabetes. The PI3K family is considered a potential immunotherapeutic target for diabetic wounds 41. MSC-derived exosomes pre-treated with pioglitazone can accelerate diabetic wound healing by enhancing angiogenesis through the activation of the PI3K/AKT/eNOS pathway 42. Future studies should focus on elucidating the specific role of individual PI3K isoforms in various phases of wound healing to examine the immune responses crucial for diabetic wound repair.

The use of MSCs as therapeutic agents represents a significant advancement in the field of biomedical research. MSCs play an essential role in the complex process of diabetic wound healing. In particular, they are involved in promoting angiogenesis, enhancing granulation, facilitating re-epithelialization, and regulating inflammatory leukocytes such as neutrophils and macrophages 43, 44. Recent studies have highlighted that the favorable effects of MSCs on wound healing predominantly rely on the involvement of small EVs 45, 46. However, the specific mechanisms through which MSC-EVs accelerate wound healing in diabetes remain unknown. MSC-induced suppression of NET formation has been shown to alleviate inflammation-related diseases 47. In this study, we used MSC-EVs, as MSCs can relay their biological effects via intercellular communication facilitated by EVs. The findings showed that MSC-EVs limited the opening of mPTP and restored the mitochondrial membrane potential balance, thereby repairing mitochondrial damage in neutrophils.

Functional mitochondria present in MSC-EVs play an essential role in alleviating mitochondrial dysfunction in neutrophils and regulating NET formation. Recent studies have shown that the delivery of functional mitochondria through MSC-EVs not only restores barrier integrity but also influences the function and phenotypic transformation of macrophages, consequently alleviating acute respiratory distress syndrome 22, 48. This study showed that MSC-EVs modulated NET formation by transferring functional mitochondria to neutrophils and influencing mitochondrial dynamics, particularly the intricate equilibrium between fusion (elongation) and fission (fragmentation), which profoundly affected mitochondrial quality in neutrophils. Mitochondrial fusion contributes to restoring mitochondrial function and enhancing metabolic activity, whereas mitochondrial fission is associated with mitochondrial apoptosis 49. Furthermore, modulation of mitochondrial dynamics can influence the functionality and polarization of neutrophils 50. In this study, treatment with MSC-EVs induced mitochondrial fusion in neutrophils; however, treatment with rhodamine 6G impaired mitochondrial function and attenuated the protective effects of MSC-EVs on mitochondria in neutrophils. Consistently, treatment with Rho-EVs attenuated wound healing accelerated by MSC-EVs in mice with diabetes.

NETs play multiple roles in regulating angiogenesis. This study showed that excessive NETosis delayed diabetic wound healing by impairing angiogenesis. NETs have been shown to trigger inflammatory angiogenesis in thromboembolic pulmonary hypertension and pulmonary arterial hypertension 51. In addition, they are involved in promoting cancer invasion and angiogenesis in both primary and metastatic sites 52. The diverse effects of NETs on angiogenesis may rely on distinct signaling pathways. NETs can enhance the proliferative and tubulogenic abilities of EC cells and the release of proangiogenic factors by triggering the TLR-4/NF-κB pathway 53. We have previously shown that NETs promote endothelial-to-mesenchymal transition by inhibiting the Hippo pathway 5. In this study, we found that NETs induced EC ferroptosis by suppressing the PI3K/AKT pathway.

Despite important findings, this study has several limitations that should be acknowledged. First, chronic wounds are a major complication of type 2 diabetes mellitus. Although the mouse model of STZ-induced diabetes used in this study represents a model of type 1 diabetes mellitus, it is commonly used to examine the NET-related delay in diabetic wound healing 10, 54, 55. Therefore, the findings of this study should be validated in other diabetic models. Second, the results of this study validated the impact of endothelial cells on wound healing regulated by NETs; however, these effects may be influenced by other cell types. This possibility cannot be dismissed given the use of a global knockout mouse model in this study. The potential crosstalk between neutrophils and other skin cells warrants further investigation. Third, MSC-EVs contain diverse biological components that may contribute to their immunomodulatory influence on NET formation. Therefore, further investigation is necessary to identify the specific components of MSC-EVs affecting NET formation. Fourth, in addition to ferroptosis, other forms of cell death involved in delayed diabetic wound healing warrant further investigation.

In conclusion, this study reveals that NET-induced ferroptosis of ECs via the PI3K/AKT pathway is responsible for impairing angiogenesis during wound healing in diabetes. The MSC-EV-mediated transfer of functional mitochondria can trigger mitochondrial fusion to preserve mitochondrial integrity in neutrophils, consequently suppressing NET formation and accelerating wound healing. This study proposes multiple targets for developing innovative therapeutic strategies for accelerating wound healing in diabetes, with one strategy involving the use of MSC-EVs.

## Supplementary Material

Supplementary methods, figures and data.
