# Supplementary material for: Mesenchymal stem cell-derived extracellular vesicles accelerate diabetic wound healing by inhibiting NET-induced ferroptosis of endothelial cells
Source: Int J Biol Sci. 2024 Jun 17;20(9):3515–29. doi: 10.7150/ijbs.97150 (PMC11234223; doi:10.7150/ijbs.97150)
Supplement: Supplementary file 1 — Supplementary methods, figures and data. [file ijbsv20p3515s1.pdf]

## Supplemental Method

### Analysis of immune cell infiltration

Analysis of the mRNA microarray datasets GSE134431 and GSE199939 aimed to ascertain the infiltration of immune cells in DFU tissues and controls through the utilization of CIBERSORT.[1] Cell types exhibiting a significant difference ($p < 0.05$) in abundance between DFU patients' tissues and controls were selected for subsequent investigation. The assessment of differentially infiltrating immune cells between DFU patients and controls involved the application of the Wilcoxon rank−sum test, and the results were visualized using the "heatmap" and "Violin plot" packages within the R software.

### Bioinformatic data collection and analysis

DFU microarray data were retrieved from the National Center for Biotechnology Information Gene Expression Omnibus (NCBI_GEO) database. A filter specifying the species type as "Homo sapiens" was employed, yielding three datasets: GSE134431, GSE199939, and GSE165816. Each dataset encompassed information from both patients with DFU and healthy controls. To identify differentially expressed mRNAs (DEmRNAs) between DFU patients and the healthy control group, analysis and comparison were conducted using the "Linear Models for Microarray Data (limma)" R package. Selection criteria for DEmRNAs included |log2 fold change| > 1 and false discovery rate (FDR)−adjusted p−value < 0.05. The biological functions of DEmRNAs in DFU were explored through Kyoto Encyclopedia of Genes and Genomes (KEGG) and gene set enrichment analysis (GSEA) utilizing the "clusterProfiler" package in the R software. Significance was determined at an

FDR−adjusted p−value < 0.05. Spearman's rank correlation analysis was executed to investigate the correlation among target genes, considering a P−value < 0.05 as statistically significant.

**Flow cytometry**

Sytox Green, recognized for its high-affinity nucleic acid staining capability, easily permeates cells with compromised plasma membranes while maintaining an inability to penetrate live cell membranes. This characteristic renders it an outstanding DNA counterstain for fixed cells. In a concise procedure, cells were incubated with Sytox, CD73, CD90, CD105, CD34, CD45, and CD44 in the dark for 30 minutes, followed by two washes and subsequent analysis via flow cytometry (Accuri C6, BD Biosciences). For the identification of mitochondrial transfer from hUC-MSC-EVs to neutrophils, MitoTracker Deep Green (Thermo Fisher Scientific, MA, US) was employed to prestain hUC-MSCs. MitoTracker Deep Green-labeled hUC-MSC-EVs were then isolated through two ultracentrifugation steps and administered to the mouse model. Intrahepatic mononuclear cells were collected at 6 hours post-transfusion, stained with APC-Cy5.5-Ly6G for 30 minutes at 4 °C in the dark, and the mean fluorescence intensity (MFI) of MitoTracker Deep Green in neutrophils was analyzed using flow cytometry. Data analysis was conducted using FlowJo (version 10) software, and the experimental process was replicated in triplicate. Statistical analyses were carried out using GraphPad Prism software.

**NET preparation**

Venipuncture was employed to collect 5 mL of blood samples from each

participant, with EDTA serving as the preservative for subsequent cell isolation. Initial separation of cells utilized Polymorphprep™ (Axis-Shield PoC, Scotland), resulting in two distinct leukocyte fractions: polynuclear cells and monocytes. For enhanced cell purity, the polynuclear fraction was sorted based on positive polymorphonuclear cells and suspended in Roswell Park Memorial Institute Medium 1640 supplemented with 5% fetal bovine serum (FBS) and 1% penicillin–streptomycin.

For NET formation, the cells were seeded at a density of $10^6$/well and stimulated with 100-nM phorbol 12-myristate 13-acetate (PMA, Beyotime, China) for 4 h at 37°C. Confirmation of NET formation was achieved through the visualization of extracellular DNA stained with SYTOX dye. Subsequently, the medium was meticulously removed, and the cell layer was gently washed with 3 mL of PBS without Ca2+ and Mg2+ ions. The PBS solution, collected after vigorous agitation, underwent centrifugation for 10 minutes at 500 g and 4°C to eliminate residual cells and debris. NET concentration was quantified using the Quant-iT PicoGreen dsDNA assay kit (P11496, Thermo Fisher Scientific, Waltham, MA, USA), and NETs were promptly utilized or stored at -80°C.

**Western blotting**

Cellular and homogenized tissue lysis were conducted using radioimmunoprecipitation assay buffer. The Omni-Easy™ Instant BCA Protein Assay Kit (ZJ102, EpiZyme, China) was employed to quantify total proteins extracted from the lysates of cells or tissues. Subsequently, separation of proteins was achieved on 4–

20% gradient gels. Primary antibodies against CD9 (1:1000, ab236630), CD63 (1:1000, ab134045), Alix (1:1000, ab275377), calnexin (1:1000, ab133615), CitH3 (1:1000, ab5103), PAD4 (1:1000, ab96758), GPX4 (1:1000, ab125066), ACSL4 (1:1000, ab155282), TFR1 (1:1000, ab214039), SLC7A11 (1:1000, ab216876), MFN1 (1:1000, ab191853), MFN2 (1:1000, ab205236), OPA1 (1:1000, ab157457), TOMM20 (1:1000, ab186735), and TSG101 (1:1000, ab 125011) were purchased from Abcam (USA). Primary antibodies against H3 (1:1000, 4499S), phosphoinositide-3-kinase (PI3K) (1:1000, 17366S), p-AKT (1:1000, 4060S), AKT (1:1000, 4685S), and DRP1 (1:1000, 8570S) were purchased from Cell Signaling Technology (CST) (USA). Antibodies against GAPDH (1:1000, 5174S) and β-actin (1:1000, 4967L) were purchased from CST, USA. Densitometry analysis was conducted using the ImageJ software (version 1.47) for quantification. The experiment was replicated three times.

**Cell culture and treatment**

Human umbilical vein endothelial cells (HUVECs) (Lonza) were cultured in ECM (#1001, ScienCell, CA, USA) supplemented with 10% FBS, 1% growth supplement (#1052, ScienCell, CA, USA) and 1% penicillin–streptomycin. HUVECs were serum-starved for 12 h and subsequently treated with the following agents in serum-free ECM for 8 h at 37°C and 5% $CO_2$: PBS (control group), NETs (500 ng/ml, NET group), NETs + DNase I (0.1 mg/mL, InvivoGen, San Diego, CA, USA), NETs + 740 Y-P (50 μg/ml, a PI3K/Akt agonist, MedChemExpress, USA), NETs + Fer-1 (1 μM, a ferroptosis inhibitor, MedChemExpress, USA).

**Cell proliferation assays**

During the 5-ethynyl-2′-deoxyuridine (EdU) assay, cells in logarithmic growth phase were seeded into 24-well plates, and the suitable concentration of EDU reagent was introduced for a 4-hour incubation period. To assess cell proliferation, the BeyoClick EdU Cell Proliferation Kit with Alexa Fluor 555 (Beyotime, China) was employed, following the manufacturer's protocol. The cells exhibiting proliferation, as indicated by EdU incorporation, displayed a vivid and consistent red fluorescence pattern. Cell nuclei were stained with Hoechst 33258 dye, which emits bright blue fluorescence.

**Animal experiments**

This study adhered to the ethical guidelines outlined in the Guide for the Care and Use of Laboratory Animals, as published by the National Institutes of Health. Mice, procured from the Shanghai Laboratory Animal Center, were housed at the Animal Science Center at Shanghai Jiao Tong University School of Medicine. The animal facility maintained a 12-hour light/dark cycle at 22°C, with two mice per cage. Experimental procedures involving mice were conducted with prior approval from the Ethical Review Board.

The protocol for establishing and evaluating skin wound healing animal models followed previously described methods.[2] The mice, including WT mice, *Padi*$4^{-/-}$ (genetic background C57BL/6J) mice, ferrostatin-1 (Fer-1, 0.8 mg/kg/d, MedChemExpress, USA) treated mice, 740 Y-P (10 mg/kg/d, MedChemExpress, USA) treated mice, MSC-EVs (100 μL of PBS containing approximately $7 \times 10^{10}$

particles) treated mice, and Rho-EVs (100 µL of PBS containing approximately 7 × $10^{10}$ particles) treated mice, were induced into a diabetic state using streptozotocin (STZ) or maintained as normoglycemic controls post-STZ buffer. Mice (7 to 9 weeks old, n = 6 per group) with random genders were randomly assigned to a treatment group and anaesthetized with isoflurane.

Two full-thickness excisional wounds were created in the shaved dorsal skin using a sterile, disposable 5-mm biopsy punch (Kai Industries, Tokyo, Japan), generating one wound on each side of the midline within a midline skin fold. Photographic documentation of the wounds occurred on days 0, 3, 7, and 14 post-wounding using an Olympus camera. Wound areas were calculated utilizing Vernier calipers to measure the wound diameter and applying the standard formula for the area of an ellipse (semi-major diameter × semi-minor diameter × Pi). Superficial blood flow in the wounds was sequentially assessed by color laser Doppler (Moor, UK), and the ratio of blood flow in the wounds was computed. The analyses of wound size and Doppler assessments were performed in a blinded fashion. Control oligonucleotides (scramble sequence) and SMAD2 siRNA (1 mg/wound) were topically administered into the wound cavity (10 mL in a vehicle of 30% pluronic F-127 gel [liquifies at 4°C but solidifies at body temperature], Sigma-Aldrich) immediately after wounding.

**Histology and image analysis**

The wounds were harvested, fixed in 4% paraformaldehyde (PFA) and embedded in paraffin. Tissue sections (5 µm) were stained with H&E stain using a

commercial kit (DAKO, Denmark) according to the manufacturer's instructions. At day 6 post-wounding, the wounds were harvested and fixed in 10% buffered formalin (16 h at 4°C, Sigma) for embedding in paraffin. Sections were deparaffinized, rehydrated, and stained with anti-CD31 (ab28364, 1:50, Abcam) and α-SMA (ab7817, 1:100, Abcam). Antibodies were used overnight at 4°C. Stained slides were photographed and analyzed in a blinded fashion using a Zeiss LSM 780 confocal microscope (Zeiss, Carl Zeiss, Germany). Vascular density in the wounds was counted after immunostaining for CD31 and α-SMA. Ten fields per section/animal (n = 6; 400 × magnification) were randomly examined and averaged to analyze the number of CD31-positive blood vessels in the wound edges. The images were analyzed using ImageJ software. Vascular density is expressed per square millimeter.

**Transmission electron microscopy**

After two washes with PBS, HUVECs underwent fixation in 1.5% glutaraldehyde for 4 hours, followed by treatment with 1% osmium tetroxide for 2 hours. Subsequently, the samples were double-distilled water washed twice (5 minutes per wash). Dehydration occurred through an ethanol gradient (50%, 70%, 90%, and 100%) and anhydrous acetone (applied three times, 15 minutes each). Saturating the samples with 1:1 and 1:2 solutions of acetone and embedding medium followed. Incubation at 37 °C for 12 hours and then at 60 °C for 48 hours ensured complete polymerization. Ultimately, 60-nm ultrathin sections underwent staining with 1% uranyl acetate and were captured using a JEM-1200EX transmission electron microscope (JEOL, Japan).

## References


1. Newman AM, Liu CL, Green MR, Gentles AJ, Feng W, Xu Y, et al. Robust enumeration of cell subsets from tissue expression profiles. Nat Methods. 2015;12(5):453-7.
2. Barcelos LS, Duplaa C, Krankel N, Graiani G, Invernici G, Katare R, et al. Human CD133+ progenitor cells promote the healing of diabetic ischemic ulcers by paracrine stimulation of angiogenesis and activation of Wnt signaling. Circulation research. 2009;104(9):1095-102.

## Supplemental figures

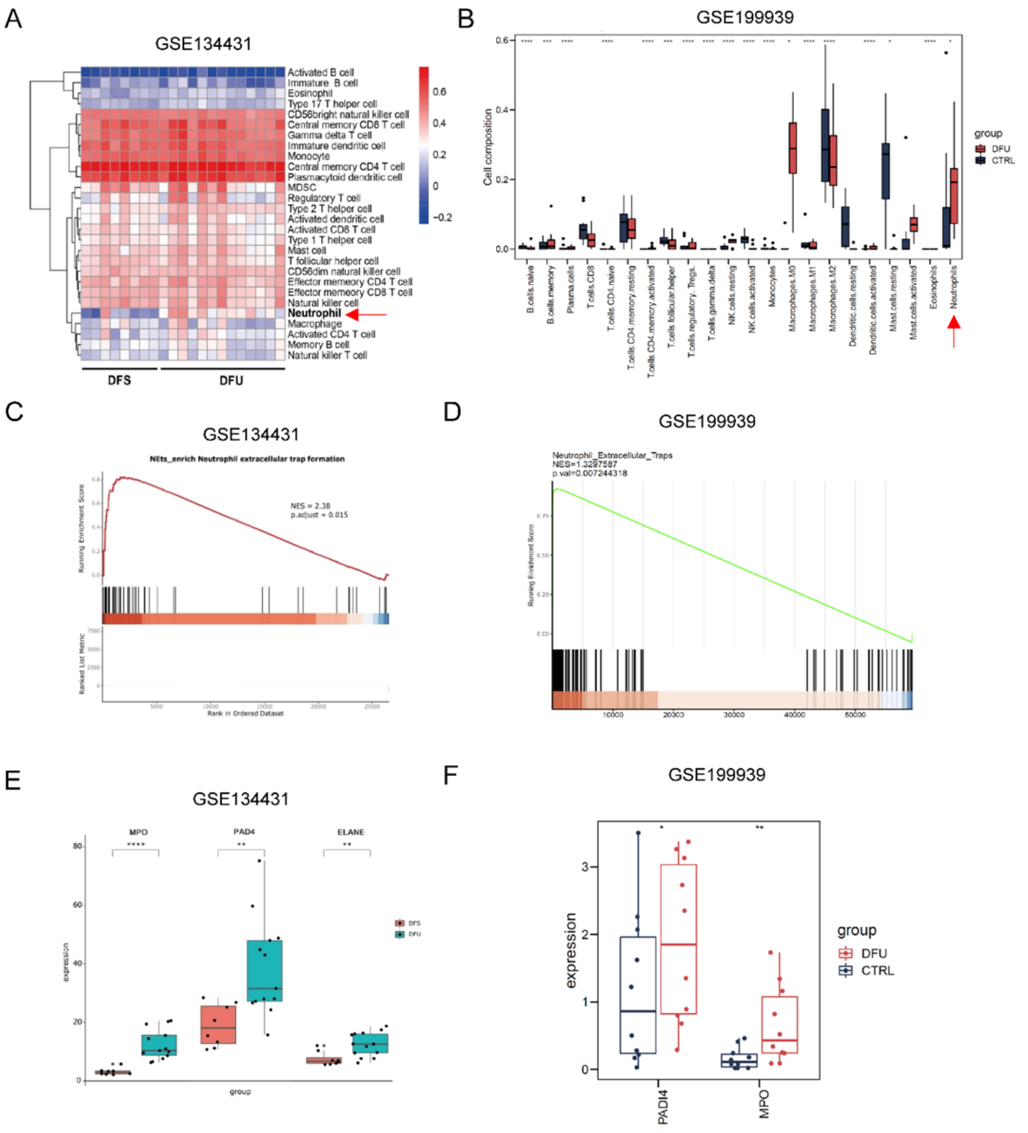


**Supplemental Figure 1. Analysis of immune cell infiltration and expression of NETs-related indicators in GEO dataset (GSE134431 and GSE199939) A-B.** significant infiltration of neutrophils in the diabetic foot ulcer (DFU) tissues; **C-F.** The gene set enrichment analysis (GSEA) and gene marker analysis revealed that NETs formation was significantly increased in the DFU tissues.

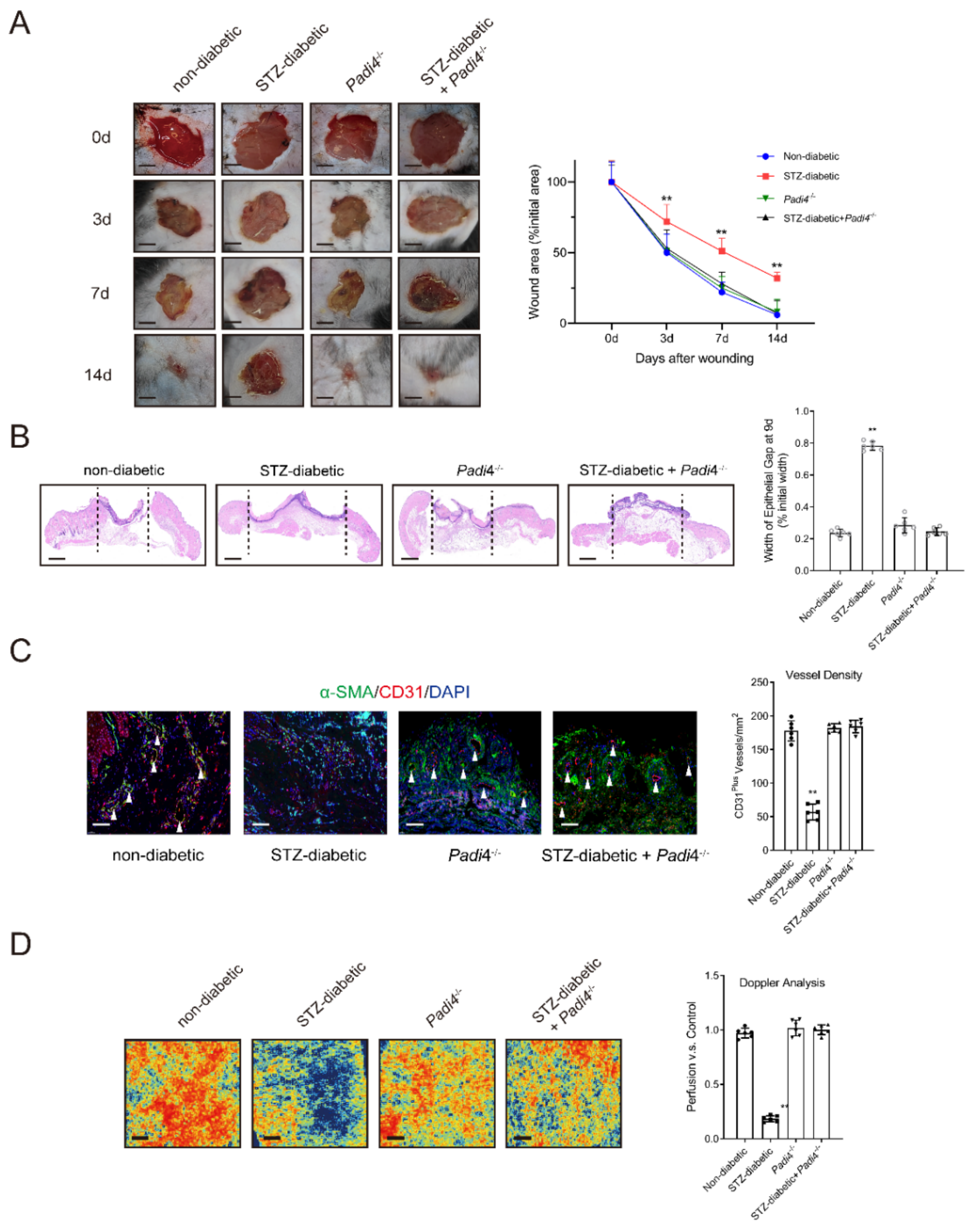


**Supplemental Figure 2. Inhibiting NET release accelerate diabetic wound healing by promoting angiogenesis A.** Left, representative images of treated wounds at 0, 3, 7, and 14 d post-wound injury in non-diabetic, STZ-diabetic, *Padi*4$^{-/-}$, and STZ-diabetic + *Padi*4$^{-/-}$ groups. Right, level of wound closure is expressed as a percentage of wound area from the initial wound area. Scare bar = 500μm. N = 3, Chi square test. **B.** Epithelial gap of wound healing on histology was evaluated in non-diabetic, STZ-diabetic, *Padi*4$^{-/-}$, and STZ-diabetic + *Padi*4$^{-/-}$ groups. The distance

between the leading edges was calculated. Scare bar = 100µm. N = 6, one-way ANOVA followed by the SNK-q post hoc test. **C.** Left, immunofluorescence staining for CD31 and a-SMA of skin wounds (400 ×) in non-diabetic, STZ-diabetic, *Padi*4$^{-/-}$, and STZ-diabetic + *Padi*4$^{-/-}$ groups. Right, quantification of vessel density expressed as CD31-positive vessels/mm$^2$. N = 6, one-way ANOVA followed by the SNK-q post hoc test. **D.** Representative color laser Doppler images taken at 5 days post-wounding. The wound perfusion was calculated as the ratio between treated and control blood flow in non-diabetic, STZ-diabetic, *Padi*4$^{-/-}$, and STZ-diabetic + *Padi*4$^{-/-}$ groups. N = 6, one-way ANOVA followed by the SNK-q post hoc test. For all subfigures: *$P < 0.05$, **$P<0.01$ compared to other groups.

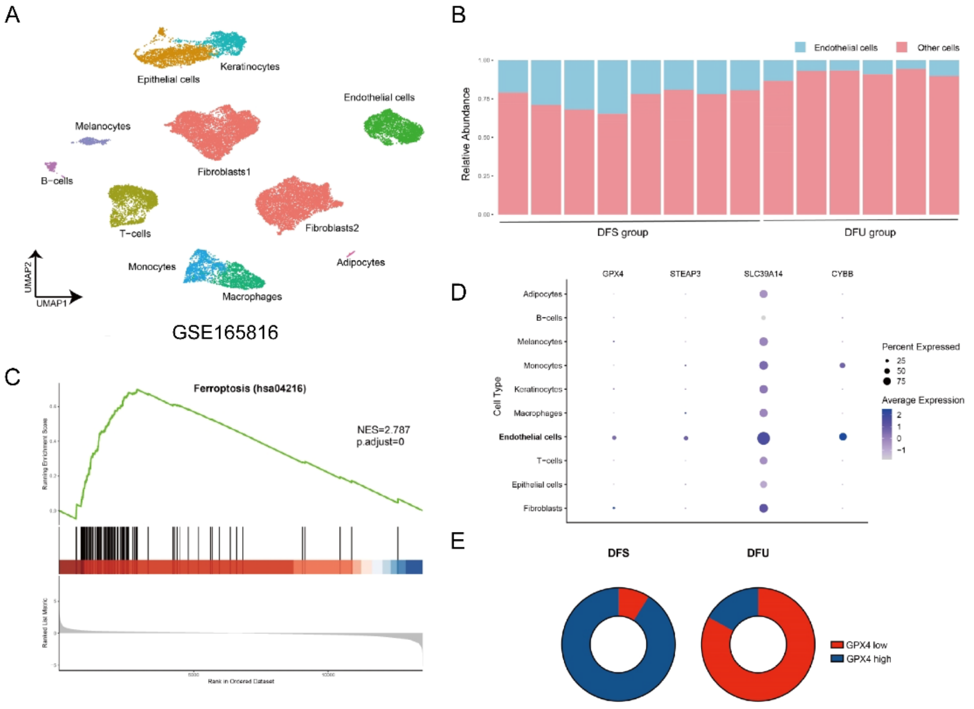


**Supplemental Figure 3. Significant ferroptosis of ECs in the diabetic foot ulcer (DFU) A-B.** The number of ECs was significantly reduced in patients with DFU compared to patients without DFU by analyzing the single-cell RNA sequencing data (GSE165816). **C-E.** GSEA and gene marker analysis showing significant up-regulation of ferroptosis markers in ECs within the DFU.

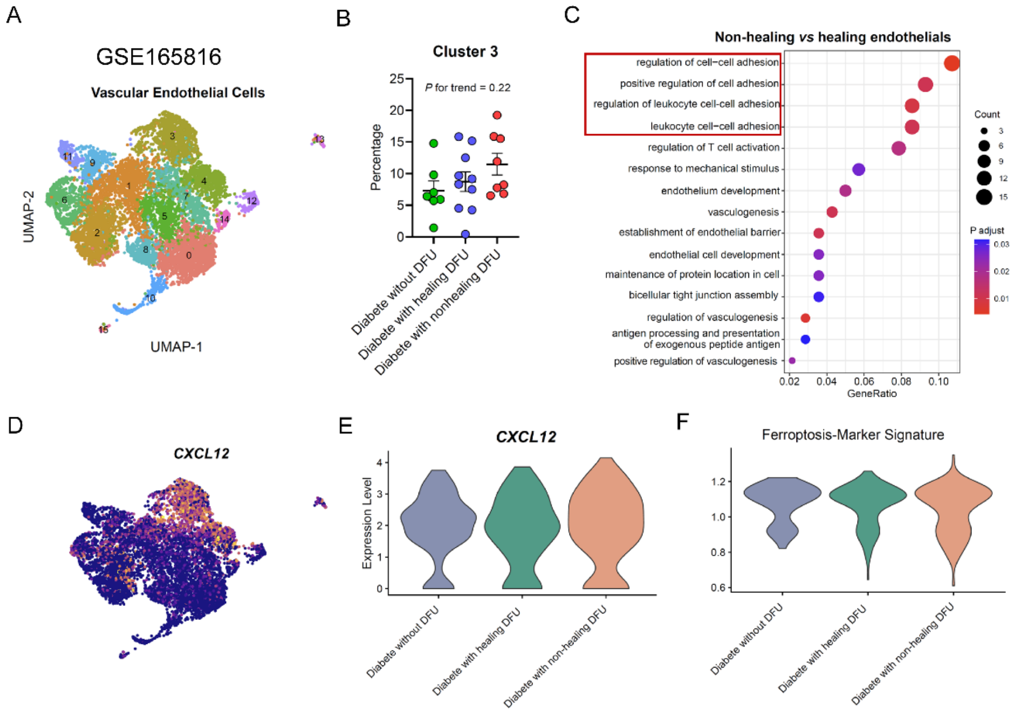


**Supplemental Figure 4. Active cell-cell adhesion between ECs with high expression of ferroptosis-related marker and neutrophils was found in diabetic wound. A-B.** The number of Cluster 3 ECs was significantly higher in diabetes with nonhealing wound than that without nonhealing wound. **C-F.** Active cell-cell adhesion was found in ECs of non-healing diabetic wound. High expression of *CXCL*12, a chemokine factor for neutrophil, and ferroptosis-related marker were found in Cluster 3 ECs.

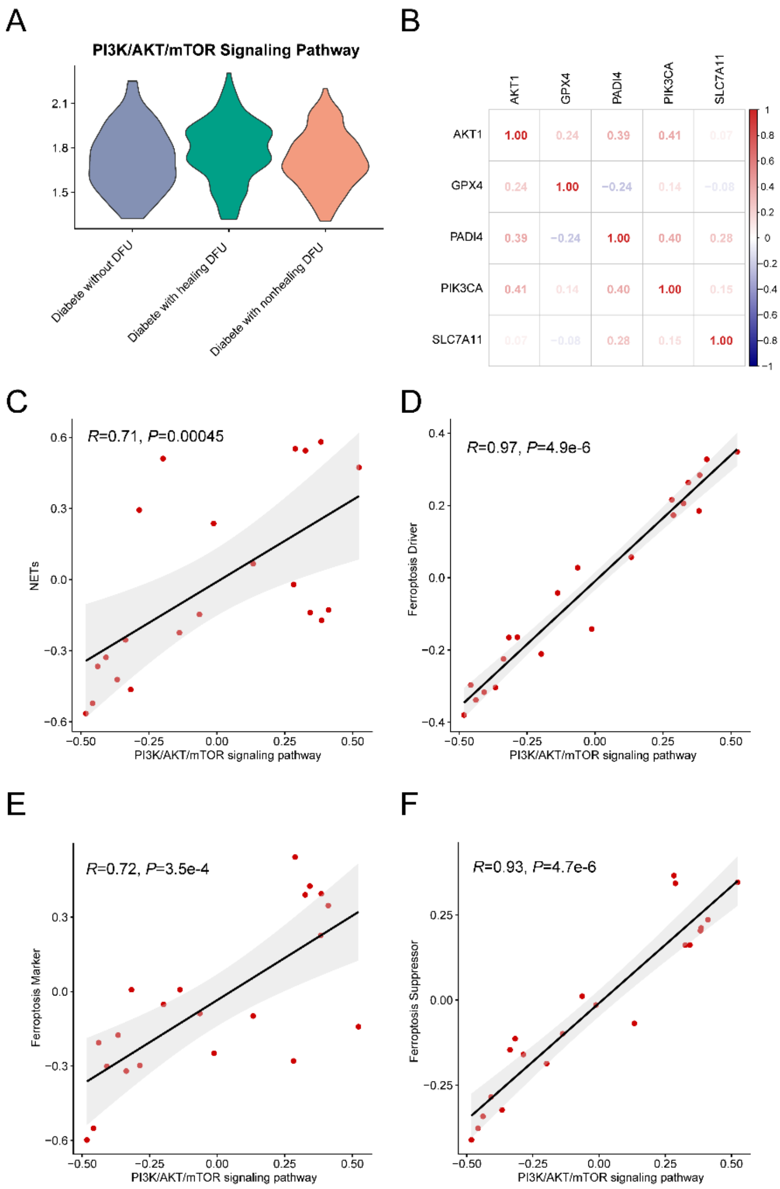


**Supplemental Figure 5. Suppression of PI3K/AKT pathway in ECs of diabetic wound in GSE134431 dataset A.** PI3K/AKT pathway was significantly suppressed in ECs of non-healing diabetic wound compared to that of healing diabetic wound. **B-F.** A correlation between PI3K/AKT pathway and NET formation or ferroptosis-related pathway in ECs of non-healing diabetic wound.

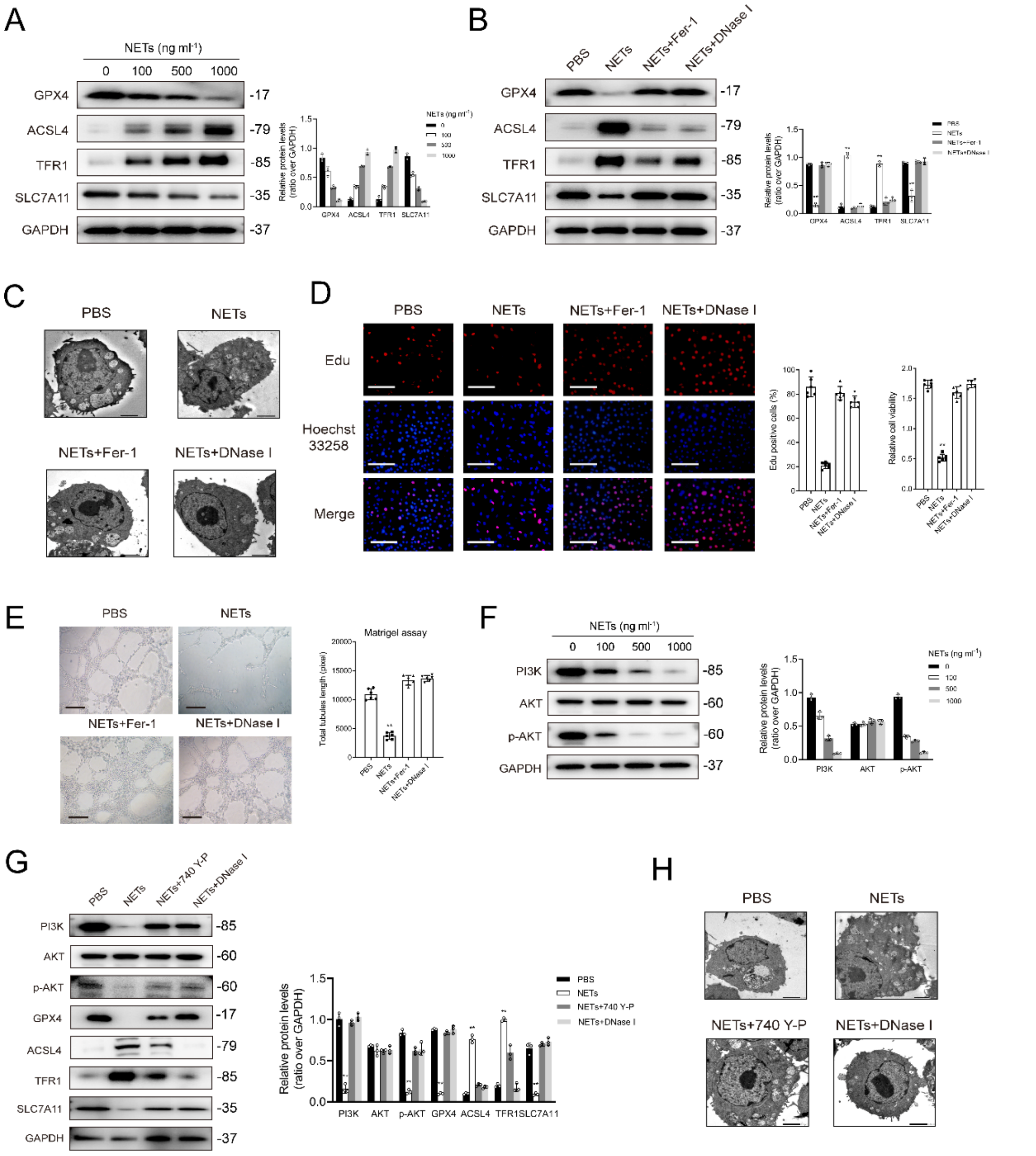


**Supplemental Figure 6. NETs-induced ferroptosis of ECs by inhibiting PI3K/AKT pathway A.** Western Blot showing the expression of ferroptosis biomarkers (GPX4, ACSL4, TFR1, and SLC7A11) in HUVECs treated with a dilution series of NETs (from 0 to 1000 ng/mL). N = 6, one-way ANOVA followed by the SNK-q post hoc test. **B.** Western Blot showing the expression of ferroptosis biomarkers in HUVECs after NET treatment with pre-incubation with Fer-1 or DNase I. N = 6, one-way ANOVA followed by the SNK-q post hoc test. **C-D.** Transmission

electron microscopy and Edu assay of HUVECs after NET treatment with pre-incubation with Fer-1 or DNase I. **E.** Representative Matrigel assay images and quantification as total tubule length values. Scale bar = 500μm. N = 6, one-way ANOVA followed by the SNK-q post hoc test. **F.** Western Blot showing the expression of AKT/PI3K pathway in HUVECs treated with a dilution series of NETs (from 0 to 1000 ng/mL). N = 6, one-way ANOVA followed by the SNK-q post hoc test. **G.** Western Blot showing the expression of AKT/PI3K pathway and ferroptosis biomarkers in HUVECs after NET treatment with pre-incubation with 740 Y-P or DNase I. N = 6, one-way ANOVA followed by the SNK-q post hoc test. **H.** Transmission electron microscopy of HUVECs after NET treatment with pre-incubation with 740 Y-P or DNase I. For all subfigures: $*P < 0.05$, $**P<0.01$ compared to other groups.

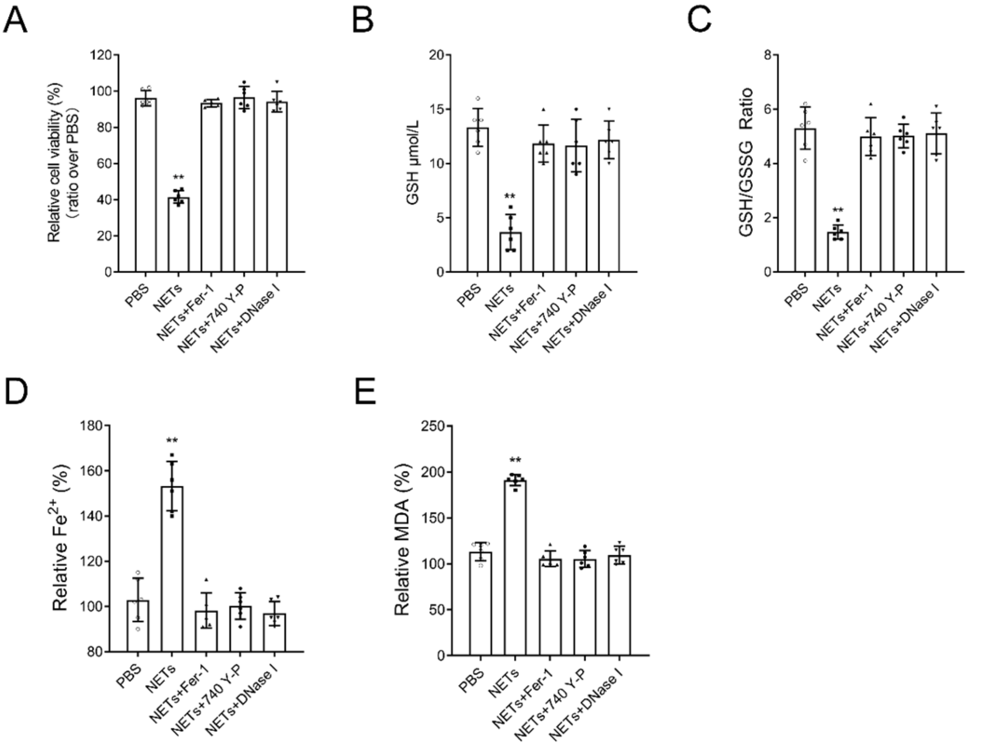


**Supplemental Figure 7. The NET-induced ferroptosis was rescued by DNase I, Fer-1, or 740 Y-P treatment A.** CCK-8 assay showing NETs-induced cell viability reduction was rescued by DNase I, Fer-1, or 740 Y-P treatment. **B-E.** NETs induced changes of ferroptosis markers (GSH, GSH/GSSG, $Fe^{2+}$, MDA) in ECs were reversed by DNase I, Fer-1, or 740 Y-P treatment. N = 6, one-way ANOVA followed by the SNK-q post hoc test. For all subfigures: $^{*}P < 0.05$, $^{**}P<0.01$ compared to other groups.

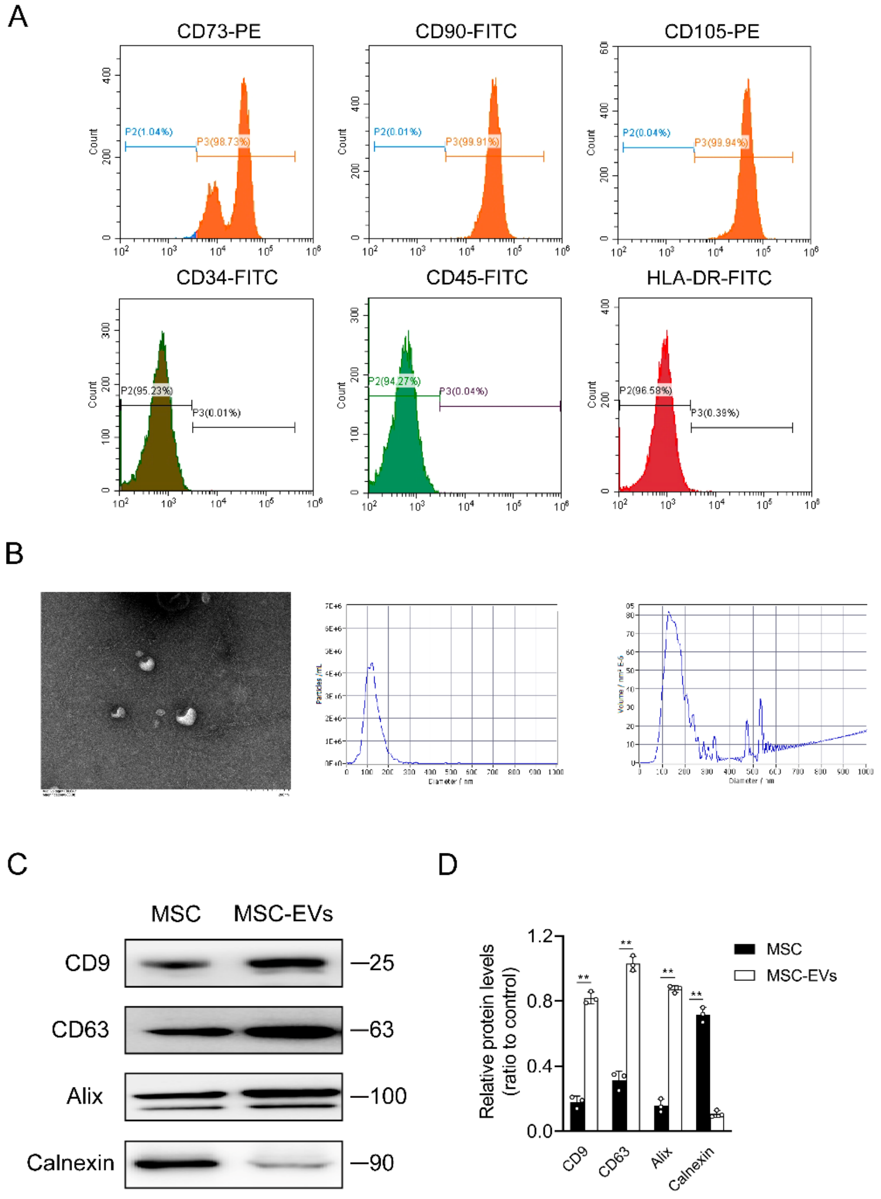


**Supplemental Figure 8. Characterization of MSC-EVs A.** Flow cytometry analysis detecting the expression of specific markers of MSCs, including CD105, CD29, CD73, CD90, CD166 and CD44. **B.** Transmission electron microscopy showing the size and spheroid morphology of MSC-EVs. Nanoparticle tracking analysis for detecting the diameter and volume of MSC-EVs. Histogram was developed from three independent measurements. **C-D.** Western blot showing the expression of specific vesicle-related markers in MSC-EVs, including CD63, CD9, Alix and

Calnexin. N = 3, one-way ANOVA followed by the SNK-q post hoc test. For all subfigures: *$P < 0.05$, **$P<0.01$ compared to other groups.

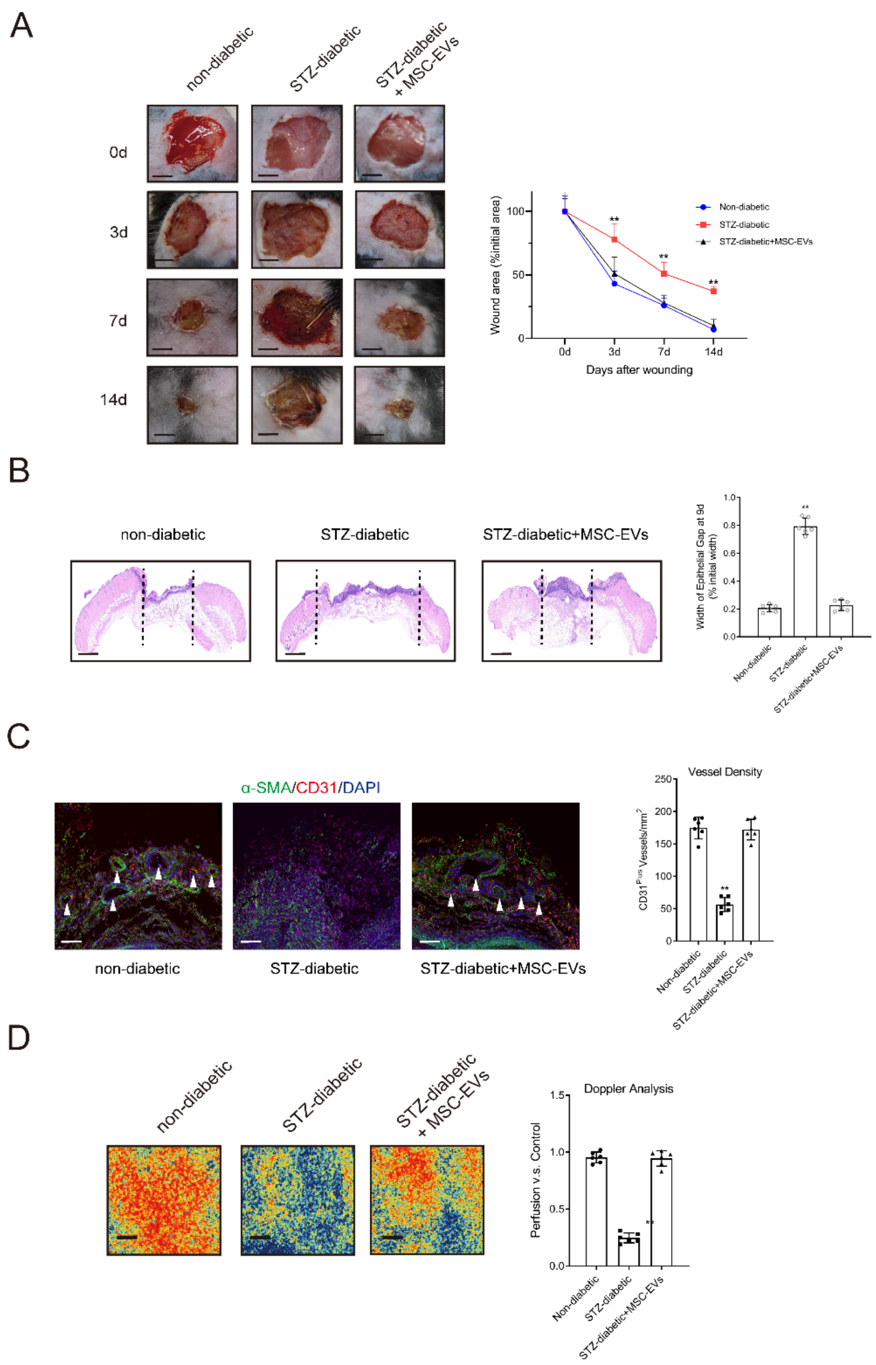


**Supplemental Figure 9. MSC-EVs can accelerate diabetic wound healing by promoting angiogenesis A.** Left, representative images of treated wounds at 0, 3, 7, and 14 d post-wound injury in non-diabetic, STZ-diabetic and STZ-diabetic + *Padi*4$^{-/-}$ groups. Right, level of wound closure is expressed as a percentage of wound area from the initial wound area. Scare bar = 500μm. N = 3, Chi square test. **B.** Epithelial

gap of wound healing on histology was evaluated in non-diabetic, STZ-diabetic, *Padi*4$^{-/-}$, and STZ-diabetic + *Padi*4$^{-/-}$ groups. The distance between the leading edges was calculated. Scare bar = 100μm. N = 6, one-way ANOVA followed by the SNK-q post hoc test. **C.** Left, immunofluorescence staining for CD31 and a-SMA of skin wounds (400 ×) in non-diabetic, STZ-diabetic, *Padi*4$^{-/-}$, and STZ-diabetic + *Padi*4$^{-/-}$ groups. Right, quantification of vessel density expressed as CD31-positive vessels/mm$^2$. N = 6, one-way ANOVA followed by the SNK-q post hoc test. **D.** Representative color laser Doppler images taken at 5 days post-wounding. The wound perfusion was calculated as the ratio between treated and control blood flow in non-diabetic, STZ-diabetic, *Padi*4$^{-/-}$, and STZ-diabetic + *Padi*4$^{-/-}$ groups. N = 6, one-way ANOVA followed by the SNK-q post hoc test. For all subfigures: *$P < 0.05$, **$P<0.01$ compared to other groups.

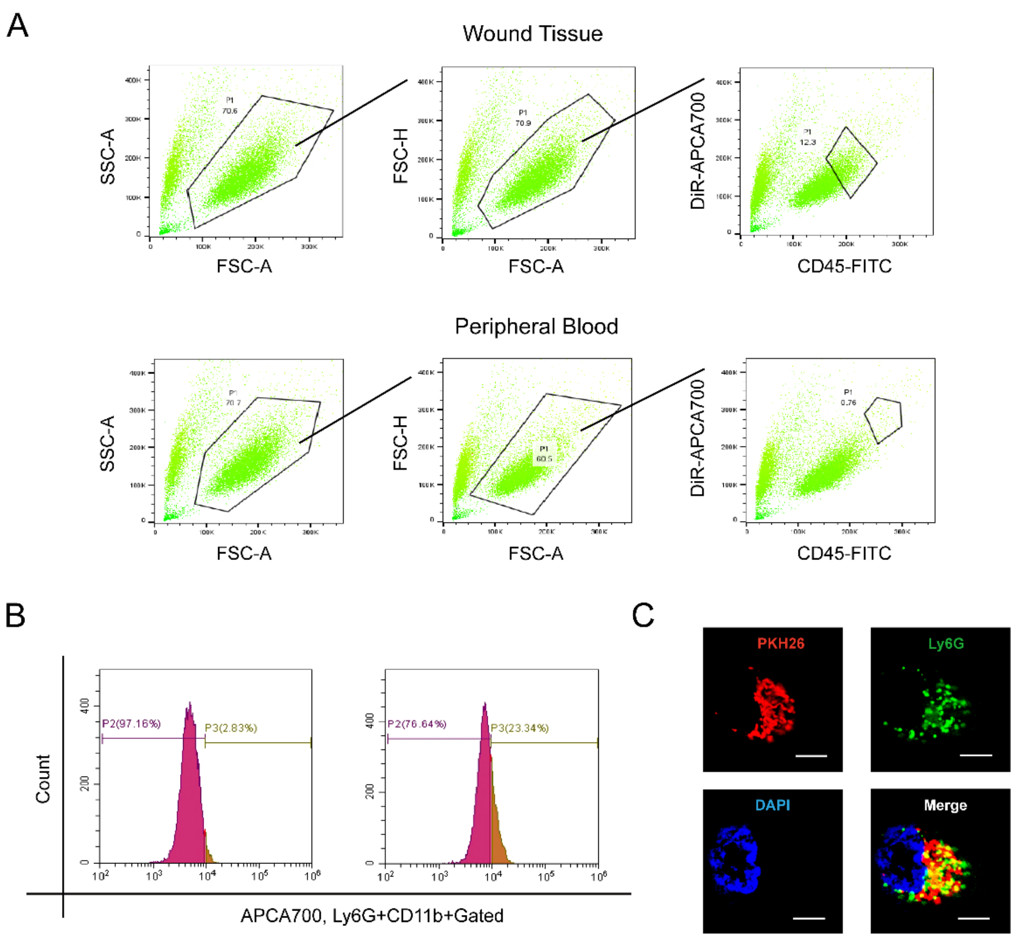


**Supplemental Figure 10. MSC-EVs were taken up by neutrophils A-B.** Flow cytometry analysis revealed that MSC-EVs were taken up by neutrophils in the wound tissue after the transfusion of DiR-labelled MSC-EVs into the mice with diabetic wound, while this phenomenon was not detected in the peripheral blood. **C.** The neutrophils were incubated with PKH26-labelled MSC-EVs for 2 h. A representative fluorescence image of MSC-EV (red) uptake in neutrophils was investigated using confocal microscopy. Scale bar=2 μm.

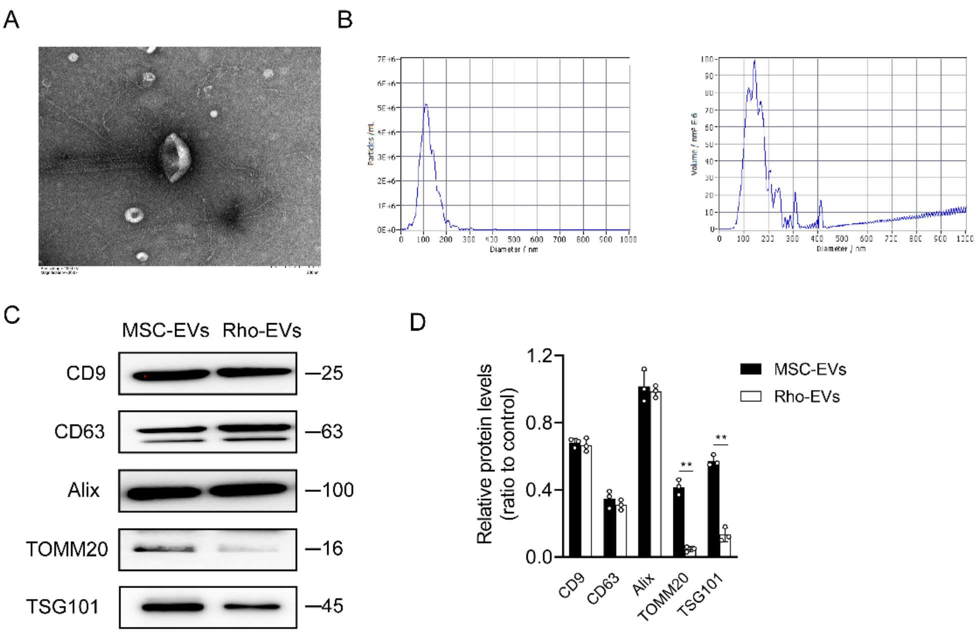


**Supplemental Figure 11. The characterization of Rho-EVs A.** Transmission electron microscopy showing the size and spheroid morphology of Rho-EVs. **B.** Nanoparticle tracking analysis for detecting the diameter and volume of Rho-EVs. Histogram was developed from three independent measurements. C. Western blot showing the expression of TOM20 and the specific vesicle-related markers in Rho-EVs, including CD63, CD9, TSG101 and ALIX. N = 3, one-way ANOVA followed by the SNK-q post hoc test. For all subfigures: *$P$ < 0.05, **$P$<0.01 compared to other groups.

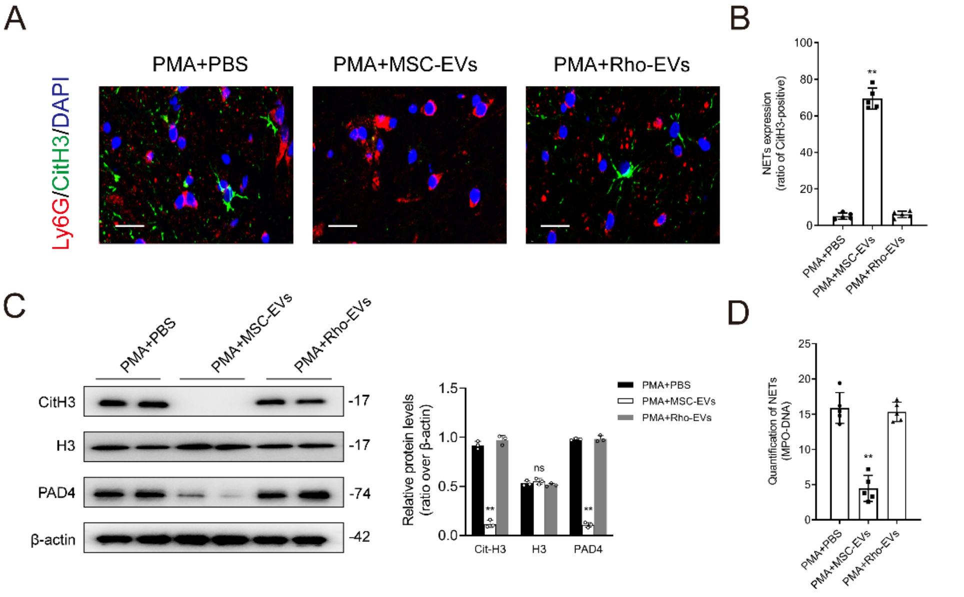


**Supplemental Figure 12. Impairing mitochondrial function reduced the inhibiting effect of MSC-EVs on NETs formation A-B.** Representative immunofluorescence images of MPO (red), CitH3 (green), and DAPI (blue) staining of neutrophils incubated with PMA+PBS, PMA + MES-EVs, and PMA + Rho-EVs for 24 h. Scale bar = 50 µm. N = 5, one-way ANOVA followed by the SNK-q post hoc test. **C.** Western blot showing Cit-H3, H3, and PAD4 in neutrophils treated with PMA+PBS, PMA + MES-EVs, and PMA + Rho-EVs. N = 4, one-way ANOVA followed by the SNK-q post hoc test. **D.** Quantification of MPO-DNA complexes in the culture supernatant. N = 5, one-way ANOVA followed by the SNK-q post hoc test. For all subfigures: *$P$ < 0.05, **$P$<0.01 compared to other groups.

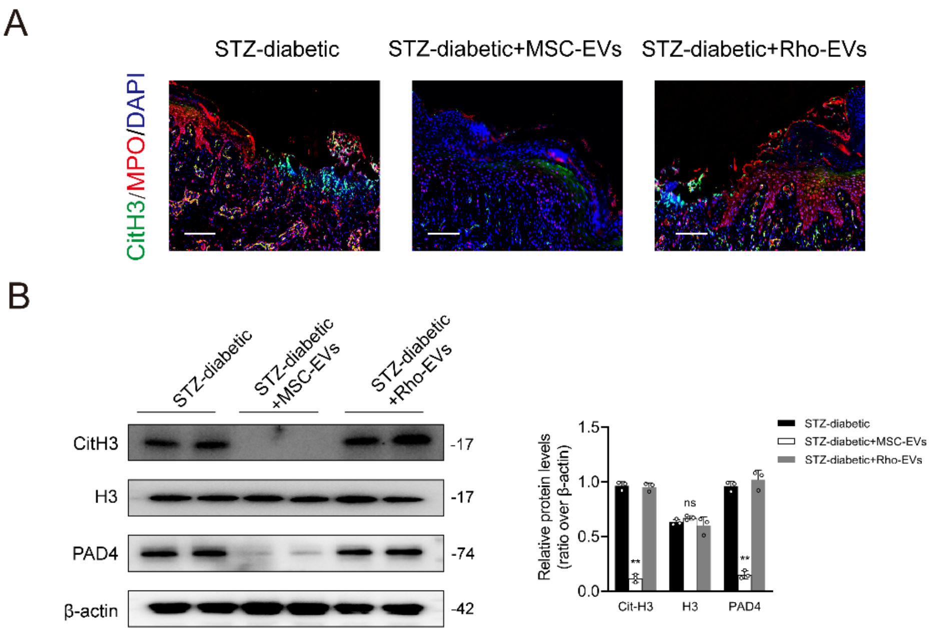


**Supplemental Figure 13. Abrogation of mitochondrial function attenuated the effect of MSC-EVs on restricting NETs formation in animal model A.** Representative images of immunofluorescence staining for MPO (red), Cit-H3 (green), and DAPI (blue) of wound tissue in STZ-diabetic, STZ-diabetic + MSC-EVs, and STZ-diabetic + Rho-EVs groups. Scale bar = 150 μm. **B.** Western blot showing Cit-H3, H3, and PAD4 expression in wound tissue from STZ-diabetic, STZ-diabetic + MSC-EVs, and STZ-diabetic + Rho-EVs groups. N = 3, one-way ANOVA followed by the SNK-q post hoc test. For all subfigures: *$P$ < 0.05, **$P$<0.01 compared to other groups.

Original Western Blots

Figure 1B

CitH3

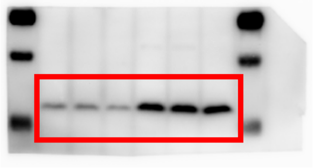

-17KDa

H3

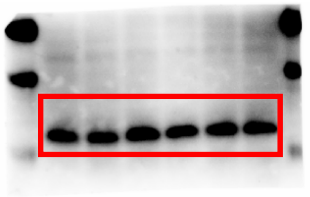

-17KDa

PAD4

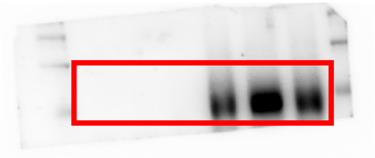

-74KDa

β-actin

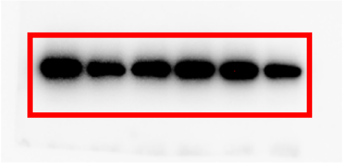

-42KDa

Figure 1D

CitH3 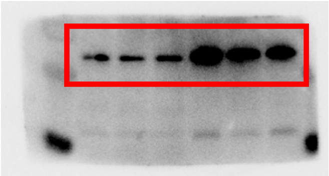 -17KDa

H3 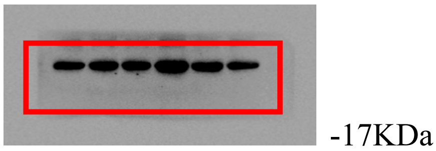 -17KDa

PAD4 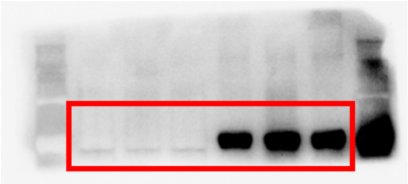 -74KDa

β-actin 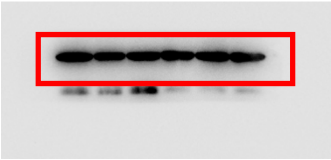 -42KDa

Figure 3B

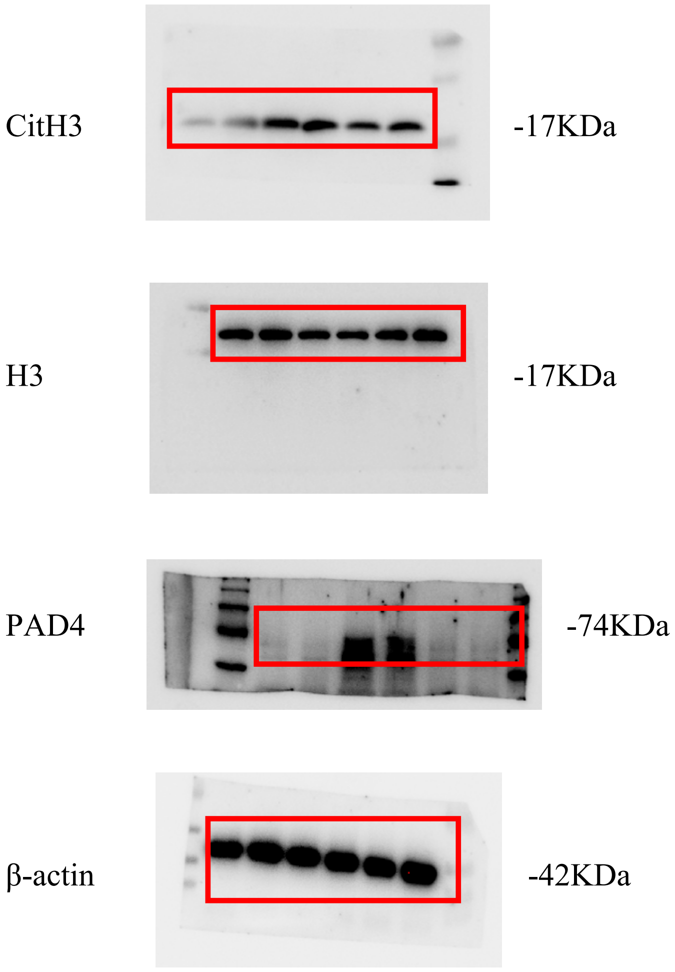

Figure 3H

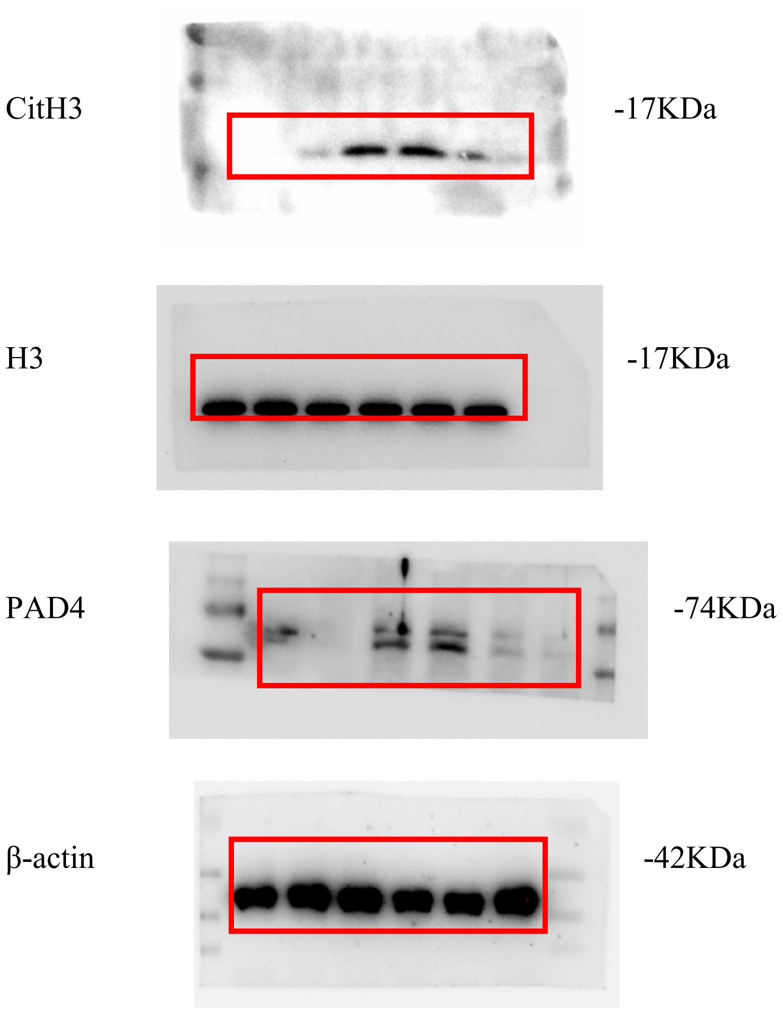

Figure 4G

Drp1

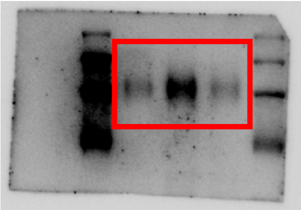

-82KDa

MFN1

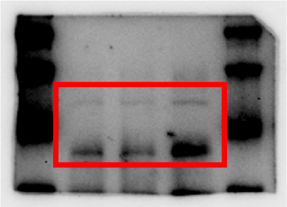

-71KDa

MFN2

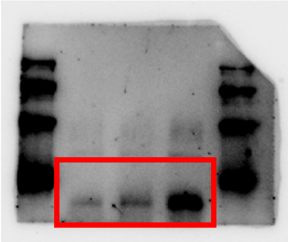

-84KDa

OPA1

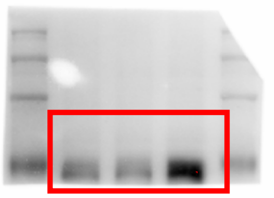

-115KDa

GAPDH

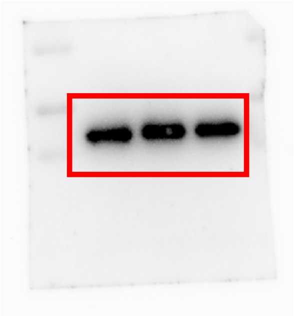

-36KDa

Figure 5G

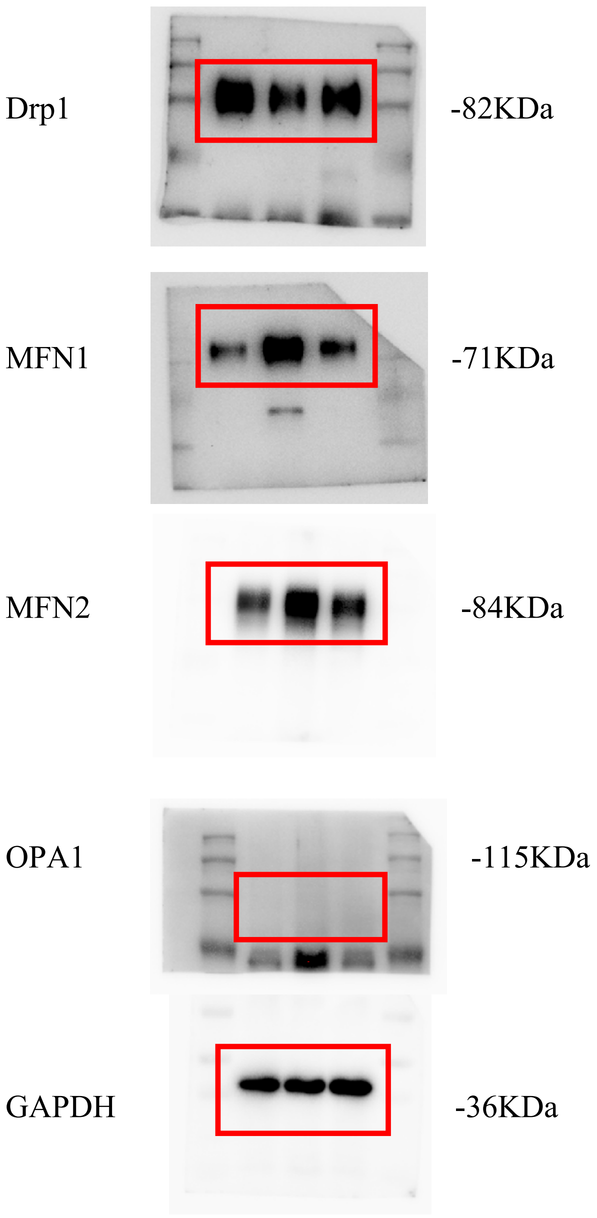

Supplemental Figure 6A

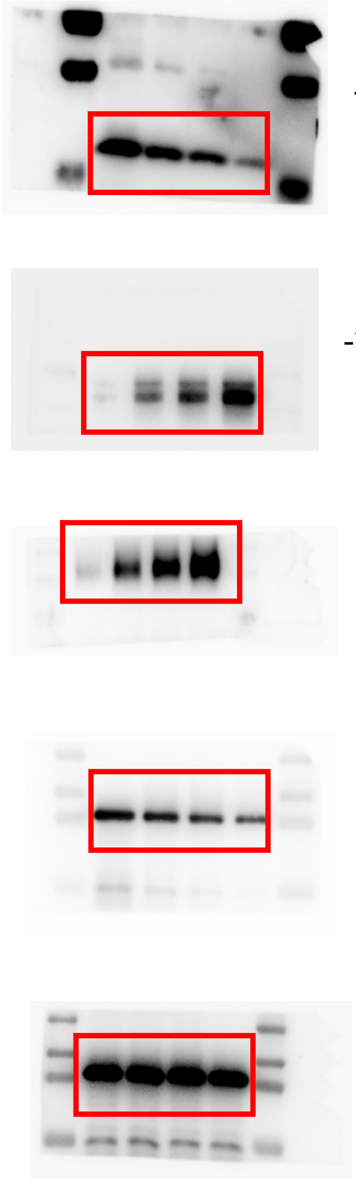

GPX4 -17KDa

ACSL4 -79KDa

TFR1 -85KDa

SLC7A11 -35KDa

GAPDH -37KDa

Supplemental Figure 6B

GPX4 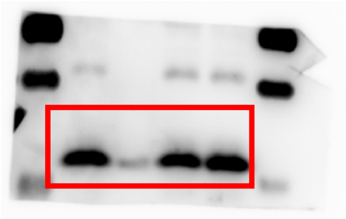 -17KDa

ACSL4 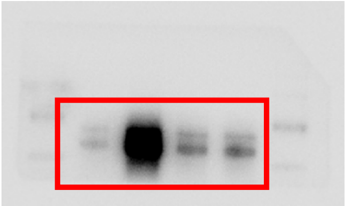 -79KDa

TFR1 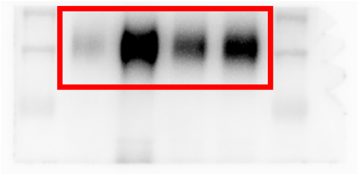 -85KDa

SLC7A11 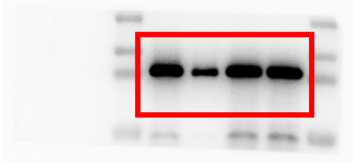 -35KDa

GAPDH 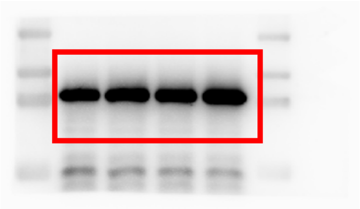 -37KDa

Supplemental Figure 6F

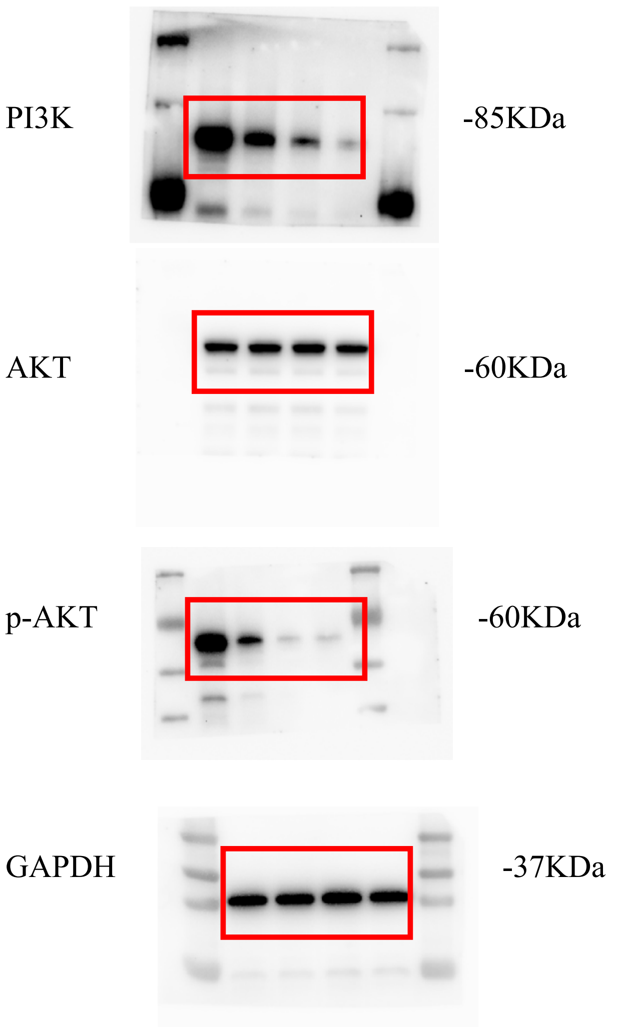

Supplemental Figure 6G

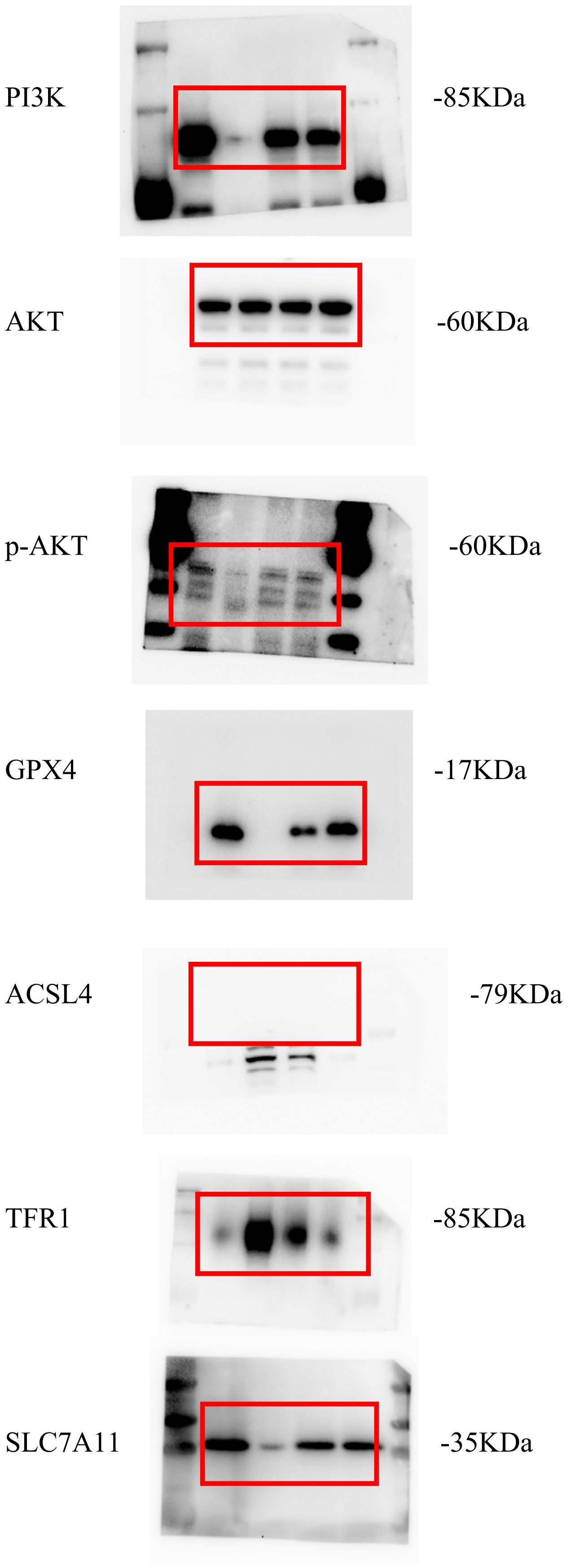

GAPDH

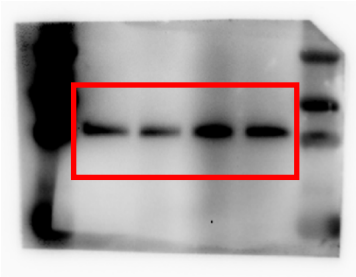

-37KDa

Supplemental Figure 8C

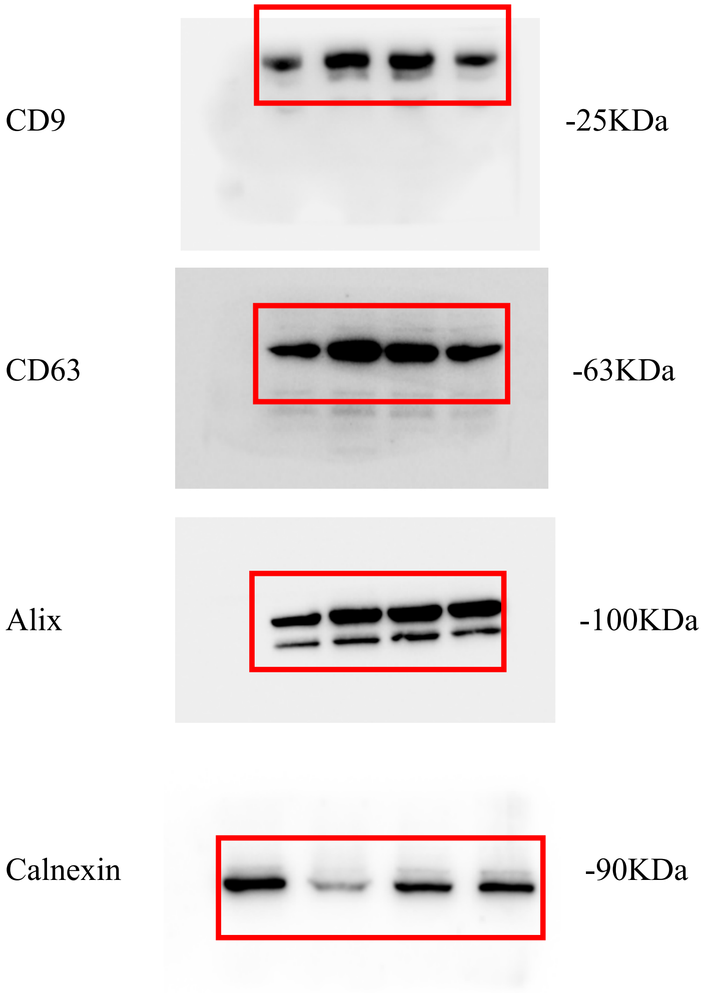

Supplemental Figure 11C

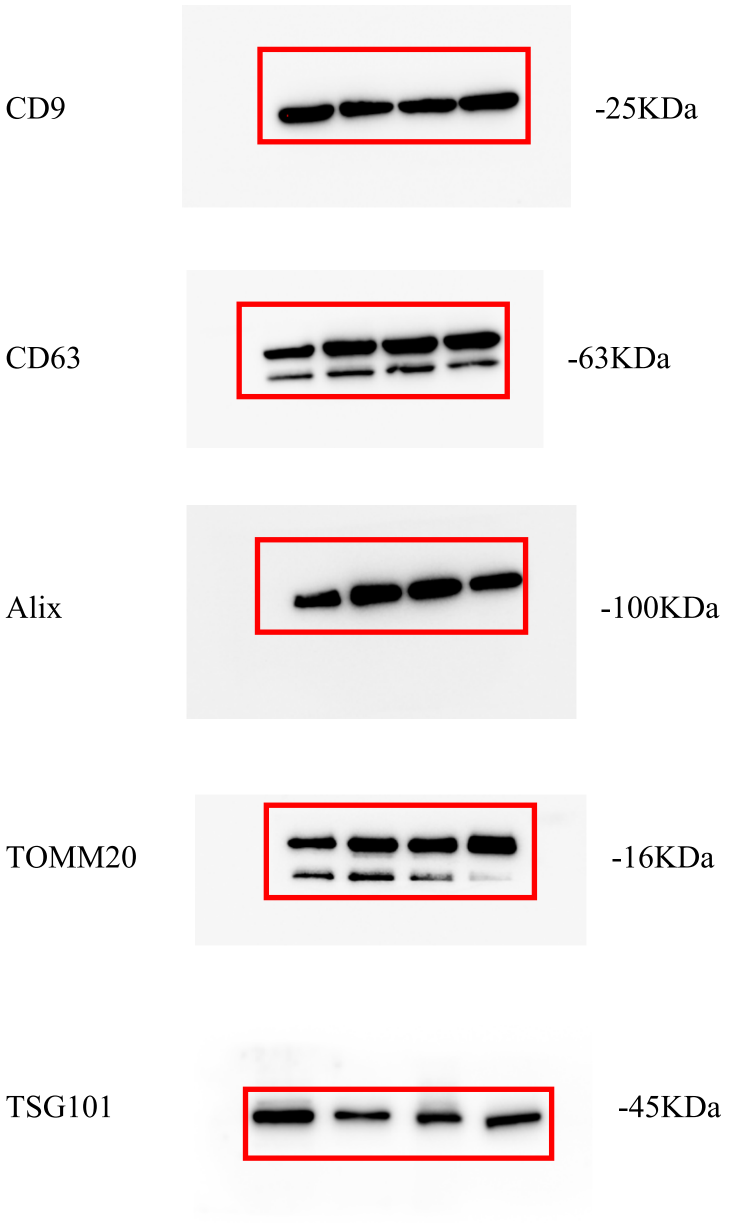

Supplemental Figure 12C

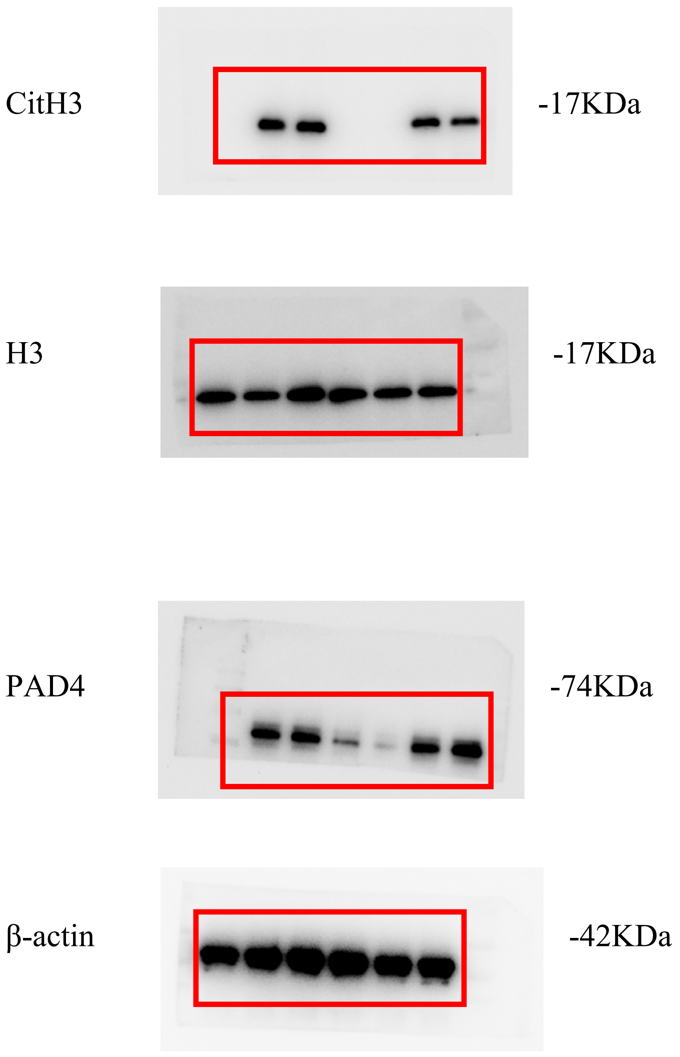

Supplemental Figure 13B

CitH3

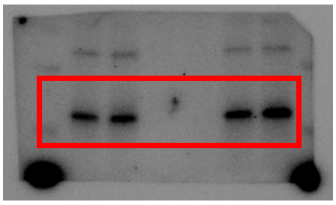

-17KDa

H3

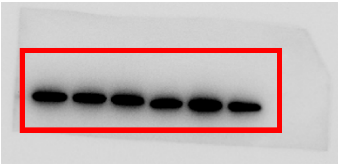

-17KDa

PAD4

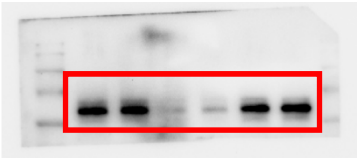

-74KDa

β-actin

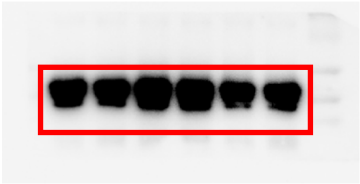

-42KDa
